# A New Basal Salamandroid (Amphibia, Urodela) from the Late Jurassic of Qinglong, Hebei Province, China

**DOI:** 10.1371/journal.pone.0153834

**Published:** 2016-05-04

**Authors:** Jia Jia, Ke-Qin Gao

**Affiliations:** School of Earth and Space Sciences, Peking University, 5 Yiheyuan Road, Beijing, 100871, China; Institute of Botany, CHINA

## Abstract

A new salamandroid salamander, *Qinglongtriton gangouensis* (gen. et sp. nov.), is named and described based on 46 fossil specimens of juveniles and adults collected from the Upper Jurassic (Oxfordian) Tiaojishan Formation cropping out in Hebei Province, China. The new salamander displays several ontogenetically and taxonomically significant features, most prominently the presence of a toothed palatine, toothed coronoid, and a unique pattern of the hyobranchium in adults. Comparative study of the new salamander with previously known fossil and extant salamandroids sheds new light on the early evolution of the Salamandroidea, the most species-diverse clade in the Urodela. Cladistic analysis places the new salamander as the sister taxon to *Beiyanerpeton*, and the two taxa together form the basalmost clade within the Salamandroidea. Along with recently reported *Beiyanerpeton* from the same geological formation in the neighboring Liaoning Province, the discovery of *Qinglongtriton* indicates that morphological disparity had been underway for the salamandroid clade by early Late Jurassic (Oxfordian) time.

## Introduction

Salamandroidea [1] are the most species-diverse group of salamanders (Caudata, Urodela), including approximately 610 extant species in seven families [2, 3]. Whether Sirenidae (sirens and their closely related fossil taxa) should be classified in Salamandroidea is a matter of debate, as recent phylogenetic results based on molecular, morphological, or combined data incompatibly place the family as the basalmost clade of Urodela [4, 5], sister clade with the Salamandroidea [6], or nested within Salamandroidea [7–13]; see [14] for a review). The members of Salamandroidea have a worldwide distribution except for Antarctica, Ethiopian realms, and the Oceanian regions of the world [15].

The suborder Salamandroidea differs from its sister clade Cryptobranchoidea [16] primarily by fusion of the angular bone to the prearticular in the lower jaw, bicapitate trunk ribs, and internal fertilization. The evolutionary history of the salamandroids can be traced back to the Mesozoic, and recent fossil discoveries from China have provided critical evidence indicating that the split of Salamandroidea from Cryptobranchoidea had taken place no later than Oxfordian time [8]. The earliest fossil record known for Salamandroidea is documented by specimens of *Beiyanerpeton jianpingensis* from western Liaoning Province [8], from fossil beds dated to the Late Jurassic at about 157 Ma [17]. The split of the two major salamander clades as a critical cladogenetic event apparently had a profound impact on shaping the evolutionary tree of salamanders as one of the three major groups of modern amphibians [18].

**Fig 1:**
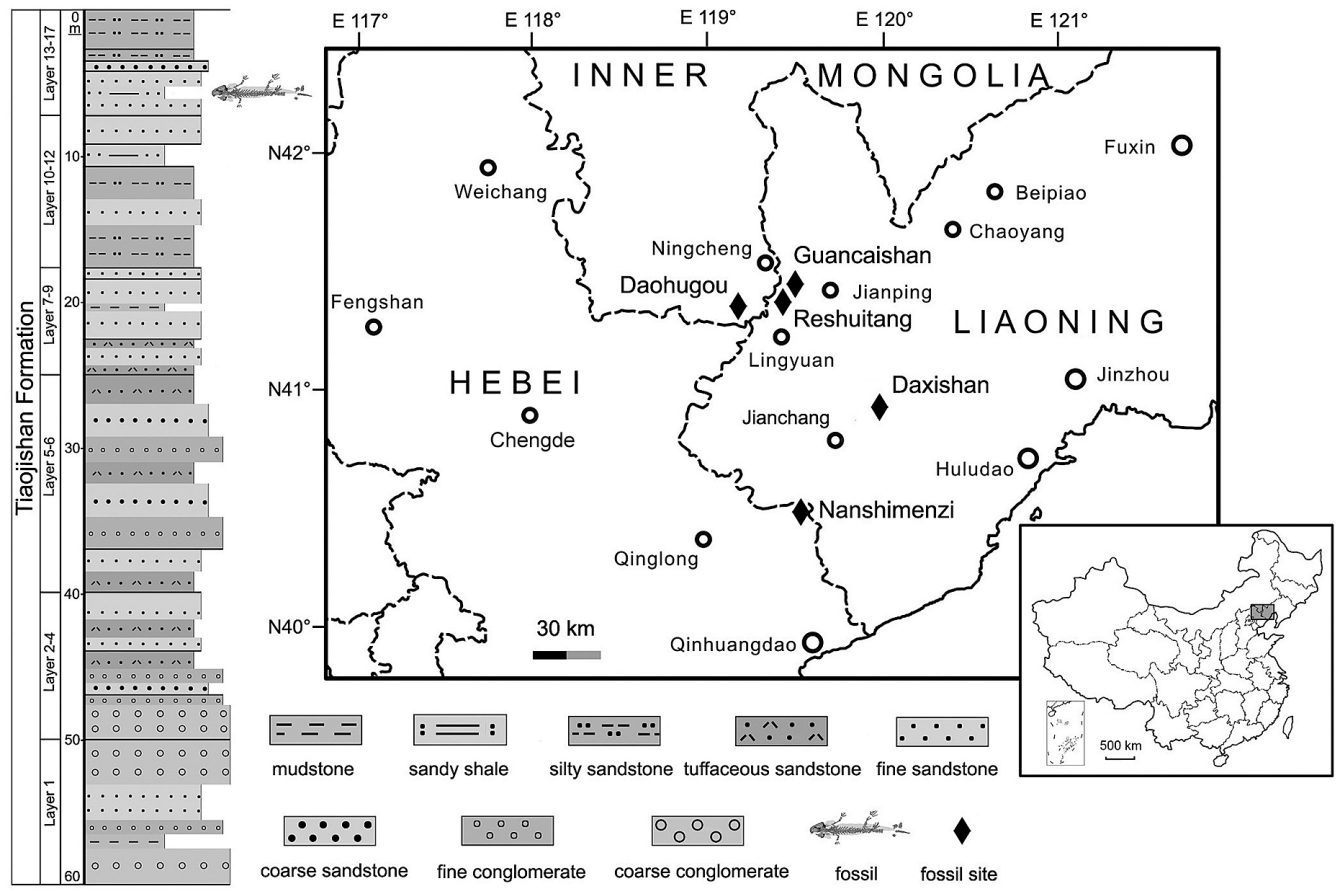
Map showing geographic location of the Nanshimenzi fossil site in relation to other Jurassic salamander fossil localities in Hebei, Liaoning, and Inner Mongolia. Stratigraphic position of the fossil beds at the Nanshimenzi site is denoted by the salamander figured on the left column.

The purposes of this paper are to describe a new salamandroid based on fossils from Upper Jurassic beds cropping out in the northeastern part of Hebei Province, northern China; to discuss the evolution of several key characters within the salamandroid clade and to assess the phylogenetic relationships of the new salamander with other salamandroids. A total of 46 specimens used in this study were collected in the summer of 2009 from a locality near the small village of Nanshimenzi, close to the provincial border with Liaoning (Fig 1). All 46 specimens are referable to the same genus and species, despite differences in body size and palatal morphology, which we interpret as ontogenetic variation. All of the specimens were collected from the same horizon of grayish-yellowish silty shales, and all have the cranial and postcranial skeletons preserved in articulation without post-mortem decomposition of the bones or disruption of them by fluvial transportation. In addition, most specimens retain evidence of soft-anatomical structures, including mineralized denticles (gill rakers) of the internal gills, impressions of the eyes and external gill filaments, and body outline. The fossil beds cropping out at the Nanshimenzi locality pertain to the Upper Jurassic (Oxfordian) Tiaojishan Formation, from which a fairly diverse vertebrate fossil fauna and a species-rich fossil flora have been revealed by recent discoveries (see Geological Setting below).

The Mesozoic fossil record of Salamandroidea is scanty, in contrast to the relatively abundant fossils of the group known from the Cenozoic. To date, the recently published *Beiyanerpeton* from China represents the only salamandroid known from the Jurassic Period [8]. The Late Jurassic *Iridotriton* from the Morrison Formation of North America was originally described as a putative salamandroid [19], but subsequently shown in a cladistic analysis to instead be a cryptobranchoid [8]. Hence, the new taxon described herein adds significantly to the Jurassic record, documenting a second salamandroid besides *Beiyanerpeton* as recently reported from the same geologic formation, but farther to the north in China. Compared to limited discoveries from the Late Jurassic, salamandroids have an improved fossil record in Cretaceous. Besides the amphiumid *Proamphiuma* in North America [20] and *Valdotriton* in Europe [21], there are a number of taxa that are viewed as possible salamandroids in North America and Europe. These are classified in Batrachosauroididae, Scapherpetontidae, and some are classified in extant families of Proteidae and Salamandridae (see [22, 23] for review). However, most of these are based on isolated vertebrae or jaws, while articulated fossils are rare. The discovery of the exquisitely preserved specimens in this collection enables a study of the detailed morphology of the new salamandroid, and a discussion on the evolution of several key characters within the salamandroid clade as presented below in the text.

### Geologic Setting and Associated Vertebrate Assemblage

**Fig 2:**
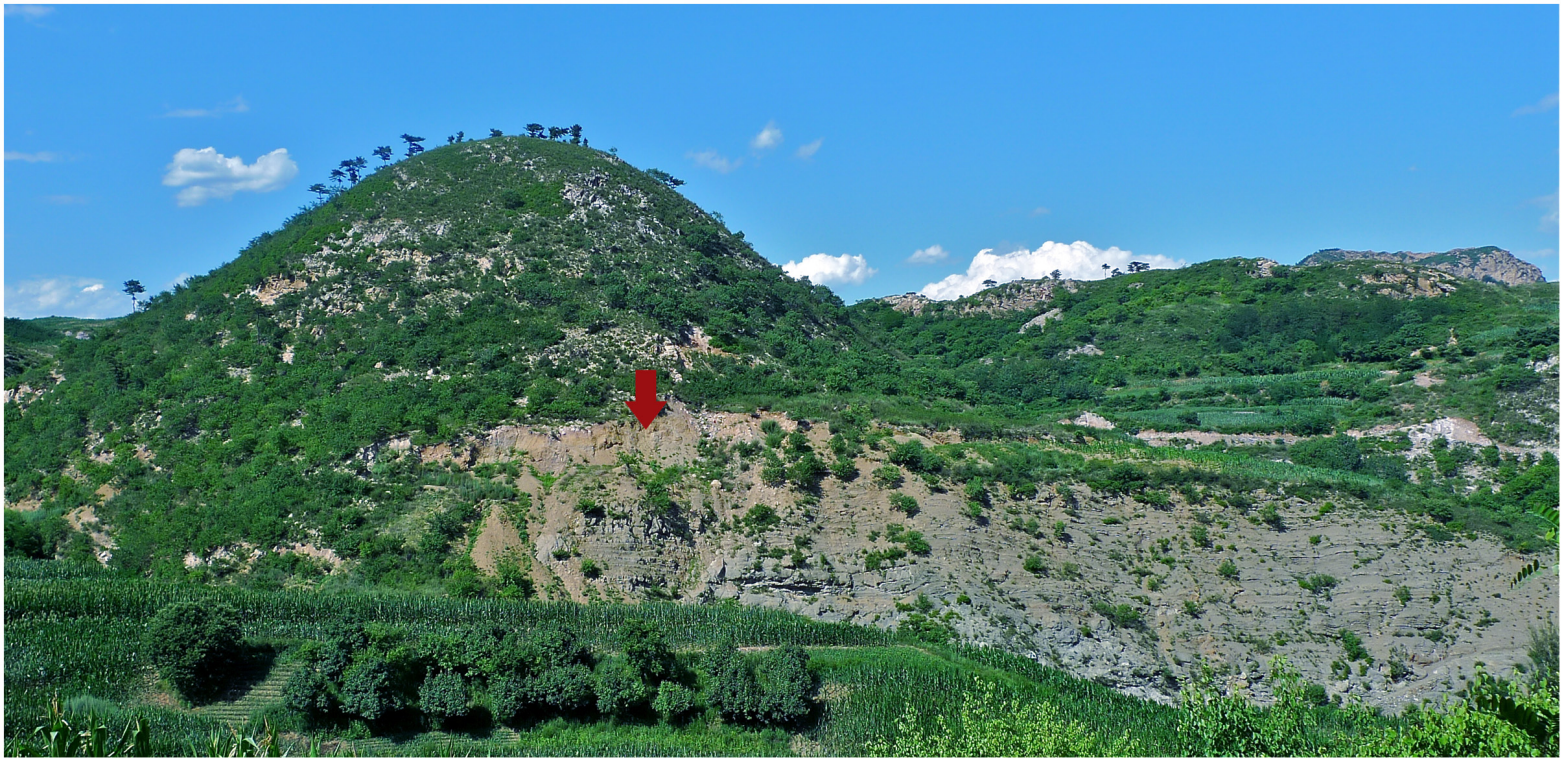
Type locality outside the Nanshimenzi Village, Qinglong Manchu Autonomous County, Qinhuangdao City, Hebei Province. Arrow pointing to the fossil beds as cropping out at the type locality.

The new salamander fossil locality (N40°31'52*"*/E119°29'11*"*) lies on a hill (Fig 2) to the north of the small village of Nanshimenzi, Qinglong Manchu Autonomous County, Qinhuangdao City, Hebei Province. The salamander fossil-bearing beds cropping out at the locality pertain to the Upper Jurassic Tiaojishan Formation; the stratigraphic equivalents of this unit in Hebei and Liaoning provinces have been termed the Lanqi Formation in some literature. The Tiaojishan (Lanqi) Formation is composed of a set of grayish purple andesite, andesite agglomerate lavas, and grayish black basalts, all intercalated with tuffaceous sandstones and ignimbrites [24]. With the thickness of the rock sequence ranging from 130 m to over 900 m, deposits of the Tiaojishan Formation crop out widely in the Western Hills of Beijing, Liaoning and Hebei provinces.

The Tiaojishan Formation has yielded high-precision ^40^Ar/^39^Ar dates of 161.8 ± 0.4 Ma near the base and 159.5 ± 0.6 Ma near the top of the formation [25, 26]. Other published radiometric dates of the formation cover a wide range, from 152 Ma to 167 Ma [17, 27–32]. Published dates compatible with those of high-precision ^40^Ar/^39^Ar dates obtained by Chang et al. [25, 26] include a ^40^Ar/^39^Ar date of 161 ± 1 Ma for the lower part of the formation at Xiabancheng, Hebei Province [27], and SIMS U-Pb Zircon dates of 158.7 ± 1.8 Ma–160.7 *±* 1.7 Ma for the Daxishan section, Jianchang Basin, western Liaoning Province [32]. These conflicting results were probably affected by specimen sampling (e.g., whole rock, biotite, feldspar and zircon), use of different dating methods (^40^Ar/^39^Ar, U-Pb SHRIMP, U-Pb LA-ICP-MS), and calculation differences [33].

No radiometric dates are directly available from the fossil beds at Nanshimenzi village. The relative age of the rock sequence has been determined by stratigraphic correlation with the Daxishan section in the Jianchang Basin [34], some 70 km northeast of Nanshimenzi village. As the Daxishan section has been recently dated at 158–160 Ma [32], the fossil beds at Nanshimenzi village can be reasonably hypothesized as falling within that time interval. In addition to the salamander fossils described in this paper, the Upper Jurassic rock sequence exposed at Nanshimenzi village and its vicinity has recently yielded other important vertebrate fossils, including several pterosaurs [35, 36], a non-avian dinosaur [37], and two mammals [38, 39].

In the past few years, important vertebrate fossil discoveries have been made at several other localities in the Tiaojishan Formation of western Liaoning Province. These include: salamander fossils from the Reshuitang and Guancaishan localities, near Lingyuan and Jianping, respectively [8, 40, 41]; and paravian dinosaur, pterosaur, and mammal fossils from the Daxishan locality near Jianchang [42–46]. Along with those from the geologically older Haifanggou (Jiulongshan) Formation (see below), fossils from the Tiaojishan Formation are components of the Yanliao Biota that immediately predates the world-famed Jehol Biota [47].

The Tiaojishan (Lanqi) Formation is disconformably underlain by the Middle Jurassic Haifanggou Formation; correlative beds of the latter in the Western Hills of Beijing are known as the Jiulongshan Formation. The Haifanggou (Jiulongshan) Formation is mainly composed of yellowish green sandstones and shales with tuffs, varying in thickness from 118 m to 533 m [24]. The Haifanggou Formation as exposed at Daohugou village, Inner Mongolia, has yielded significant vertebrate, invertebrate, and plant fossils that are important components of the Yanliao Biota (= Daohugou Biota in Sullivan et al. [48]). It must be stressed that essentially all the fossils described in the literature so far (e.g., the mammal *Magaconus* Zhou et al. [49]) from the Daohugou beds (sensu stricto) are from the Haifanggou Formation, where published radiometric dates for the “Daohugou beds” at the Daohugou section [17, 25, 28, 30, 50] are derived from the overlying Tiaojishan Formation. Dating of the Haifanggou (Jiulongshan) Formation is only available by analysis of rock samples from the Chengde area in Hebei Province ([51]: U-Pb 163.4 ± 1.1 Ma) and from the Beipiao Basin in Liaoning Province ([26]: ^40^Ar/^39^Ar 166.7 ± 1 Ma).

## Materials and Methods

All specimens of this study are deposited in the Peking University Paleontological Collections (PKUP), Beijing, China, and are accessible by qualified professionals. No permits were required for the described study. PKUP V0226 is designated as the holotype because it is a large specimen with well-preserved cranial and postcranial skeleton, and it shows diagnostic features in the palate. Specimens were manually prepared using fine needles under a Leica MZ 16 microscope. Selected specimens (PKUP V0226, V0228, V0254) were scanned at China University of Geosciences (Beijing) using a High-Resolution X-ray CT scanner (Nikon XT H 320 LC). Scanning of specimens was performed using no filter, at voltage of 180 kV and a current of 105 uA for PKUP V0226, 170 kV and 63 uA for PKUP V0228, 170 kV and 47 uA for PKUP V0254. Voxel size (mm) is 0.066624×0.066624 for PKUP V0226, 0.118609×0.118609 for PKUP V0228, 0.078569×0.078569 for PKUP V0254. These specimens were scanned along the coronal axis for a total of 1998 slices at an image resolution of 2000×2000. Segmentation and 3D reconstruction of the specimens are made by using VG Studio Max 2.2 (Volume Graphics, Heidelberg, Germany). Line drawings of anatomical structures were prepared using Adobe Photoshop (CS4).

All measurements were obtained from manually held calipers. Total length (TL) refers to the distance between the tip of the snout and the posterior extremity of the last caudal vertebra; snout-pelvic length (SPL) is the measurement from the snout tip to the posterior end of the pelvis; skull length (SKL) is the maximum dimension from snout tip to the posterior extremity of the occipital condyles; skull width (SKW) is the greatest distance between cranio-mandibular joints. The mean of three independent measurements of each parameter was taken to minimize errors. As most of the specimens have part of the tail missing, TL is obtainable from few specimens (PKUP V0233, V0236, V0263, V0266). The SPL from 27 measurable specimens ranges from 45.53 mm to 155.71 mm, with PKUP V0226 (holotype) the largest, and PKUP V0261 the smallest. The remaining 19 specimens have the snout or pelvis partly missing, making the SPL unobtainable. Differences in SPL or SKL in combination with extent of ossification of skull elements were used to estimate the relative age of individual specimens. In addition, ossification of the septomaxilla, presence of posterolateral processes of basibranchial II and extensive ossification of epiphysis in long bones were used as criteria to distinguish adults from juveniles.

We follow Milner [52] in using the term ‘Caudata’ for total-group, and the term ‘Urodela’ for crown-group, salamanders. However, we accept the commonly used classification of Cryptobranchoidea and Salamandroidea as two suborders within Urodela, regardless of the controversial position of Sirenidae as a basal urodele clade or an ingroup clade of Salamandroidea. Anatomical descriptions were mainly based on observations from the holotype, but were also supplemented with information from referred specimens.

### Nomenclatural Acts

The electronic edition of this article conforms to the requirements of the amended International Code of Zoological Nomenclature, and hence the new names contained herein are available under that Code from the electronic edition of this article. This published work and the nomenclatural acts it contains have been registered in ZooBank, the online registration system for the ICZN. The ZooBank LSIDs (Life Science Identifiers) can be resolved and the associated information viewed through any standard web browser by appending the LSID to the prefix “http://zoobank.org/”. The LSID for this publication is: urn:lsid:zoobank.org:pub:582E2169-76E0-4592-8CEB-94AF6D5C3353. The electronic edition of this work was published in a journal with an ISSN, and has been archived and is available from the following digital repositories: PubMed Central (http://www.ncbi.nlm.nih.gov/pmc), LOCKSS (http://www.lockss.org).

### Systematic Paleontology

Class Amphibia Linnaeus, 1758

Subclass Lissamphibia Haeckel, 1866

Superorder Caudata Scopoli, 1777

Order Urodela Duméril, 1806

Suborder Salamandroidea Dunn, 1922

Family Incertae Sedis

### Genus *Qinglongtriton* gen. nov.

urn:lsid:zoobank.org:act:DE89F7C6-1870-4B3E-A4BD-8C2BB208D193

### Qinglongtriton gangouensis gen. et sp. nov.

urn:lsid:zoobank.org:act:B8CFC9E4-051C-4DD9-8C9F-0EC6AF0C7C0A

(Figs 3–9)

**Fig 3:**
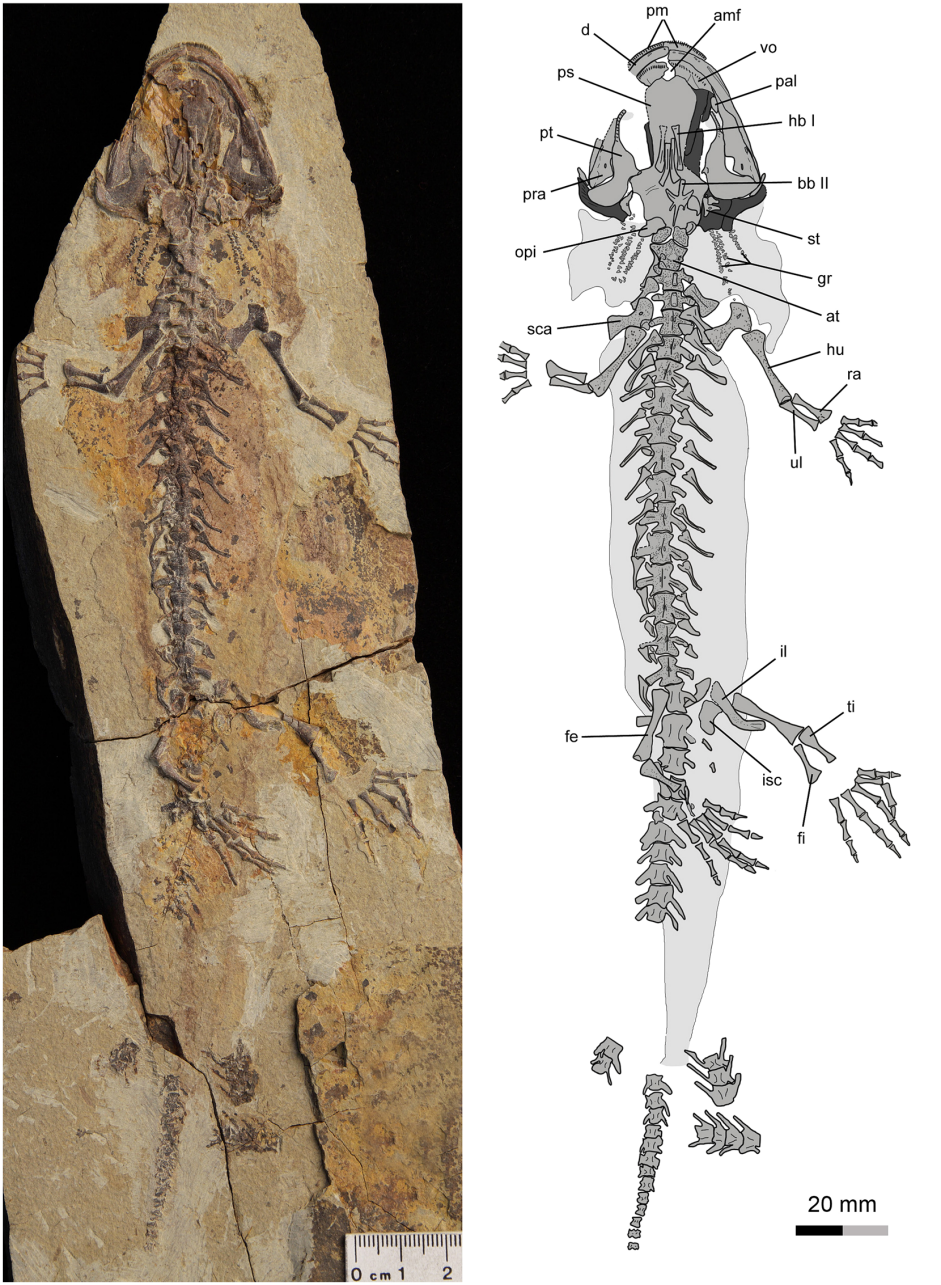
Holotype of *Qinglongtriton gangouensis* (PKUP V0226). Photograph (left) and line drawing (right) of incomplete skeleton in ventral view.

**Fig 4:**
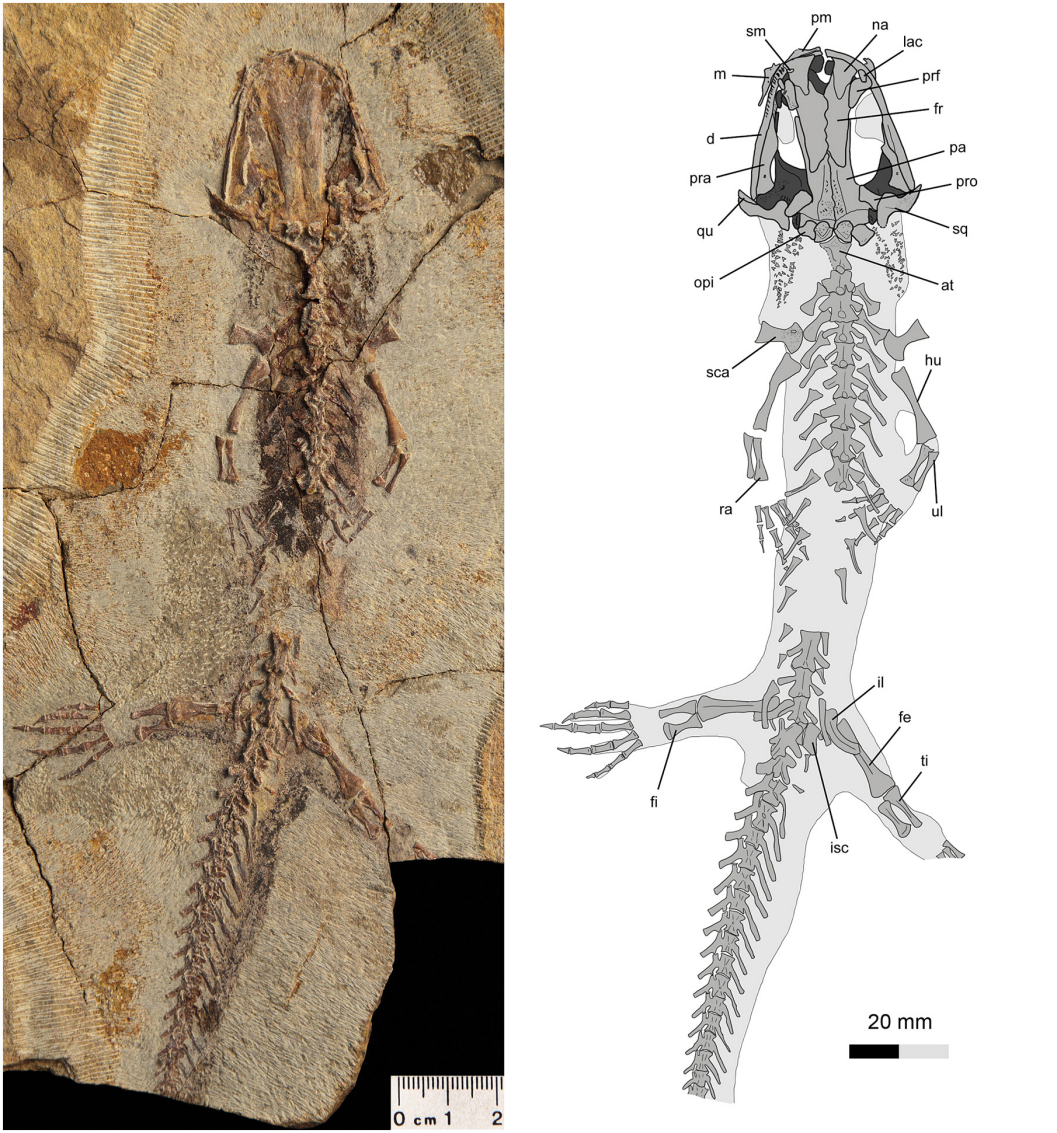
Referred specimen of *Qinglongtriton gangouensis* (PKUP V0228). Photograph (left) and line drawing (right) of incomplete skeleton in dorsal view.

**Fig 5:**
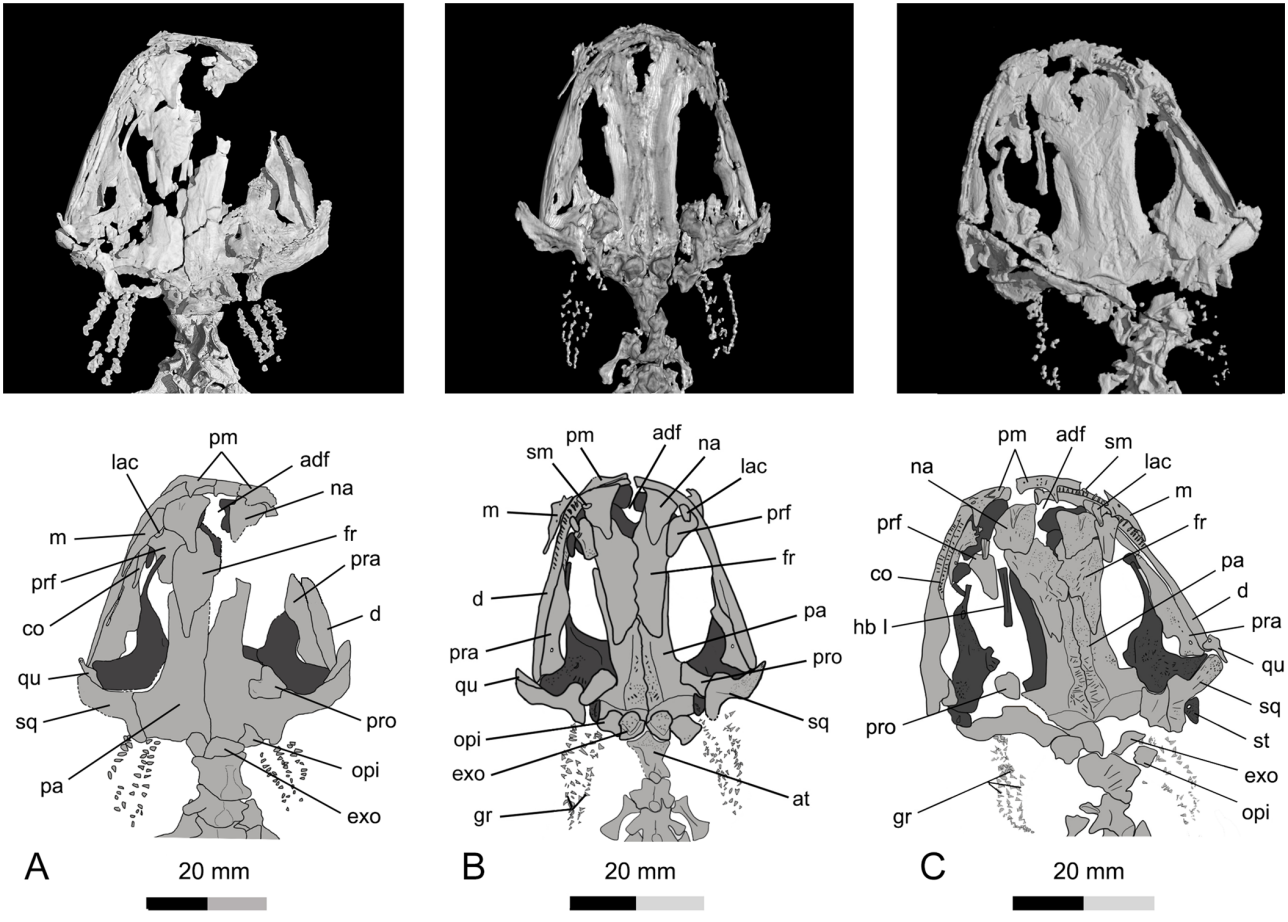
Skull of *Qinglongtriton gangouensis* in dorsal view. CT images (top) and line drawings (bottom). A, PKUP V0226, holotype skull; B, PKUP V0228, referred specimen; C, PKUP V0254, referred specimen.

**Fig 6:**
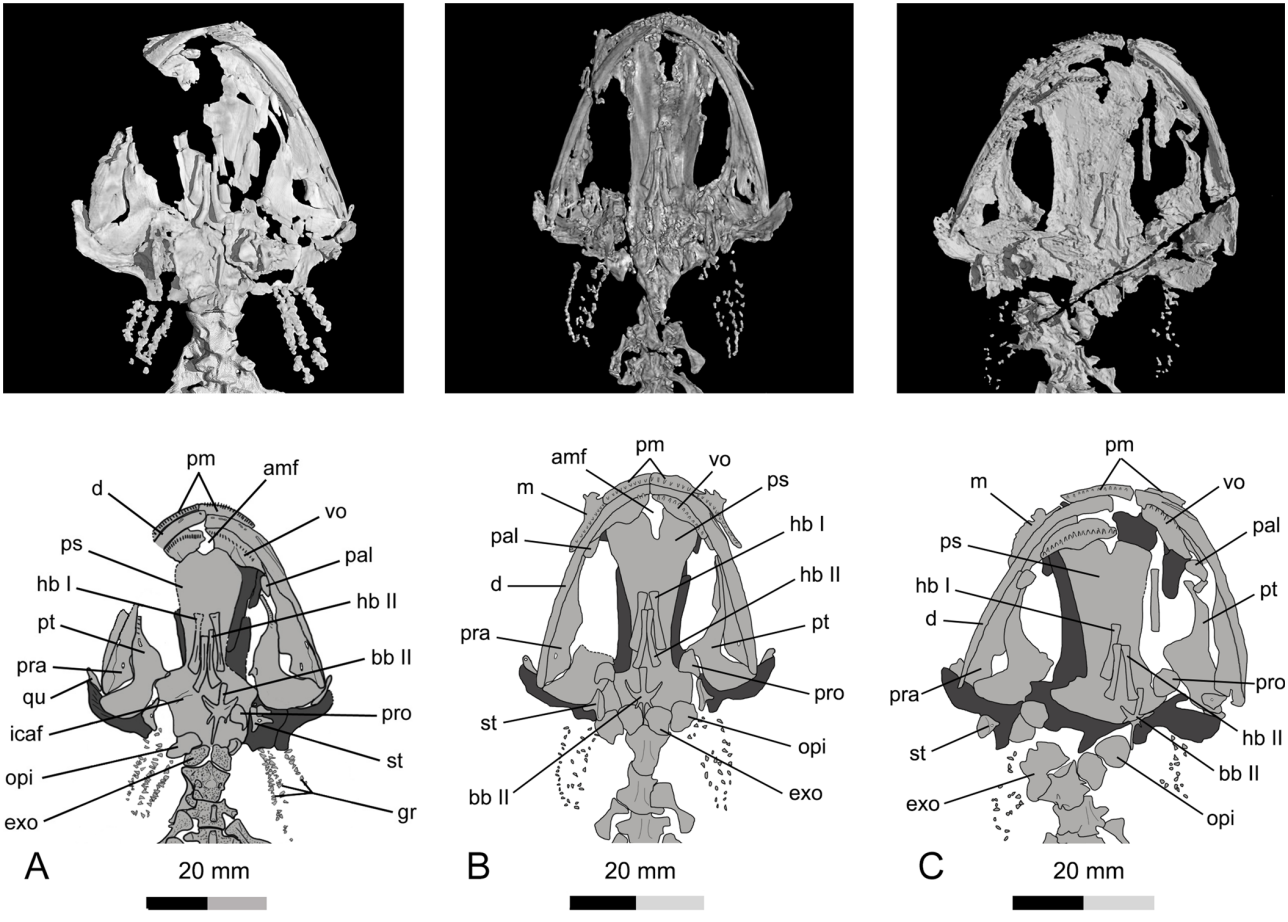
Skull of *Qinglongtriton gangouensis* in palatal view. CT images (top) and line drawings (bottom). A, PKUP V0226, holotype skull; B, PKUP V0228, referred specimen; C, PKUP V0254, referred specimen.

**Fig 7:**
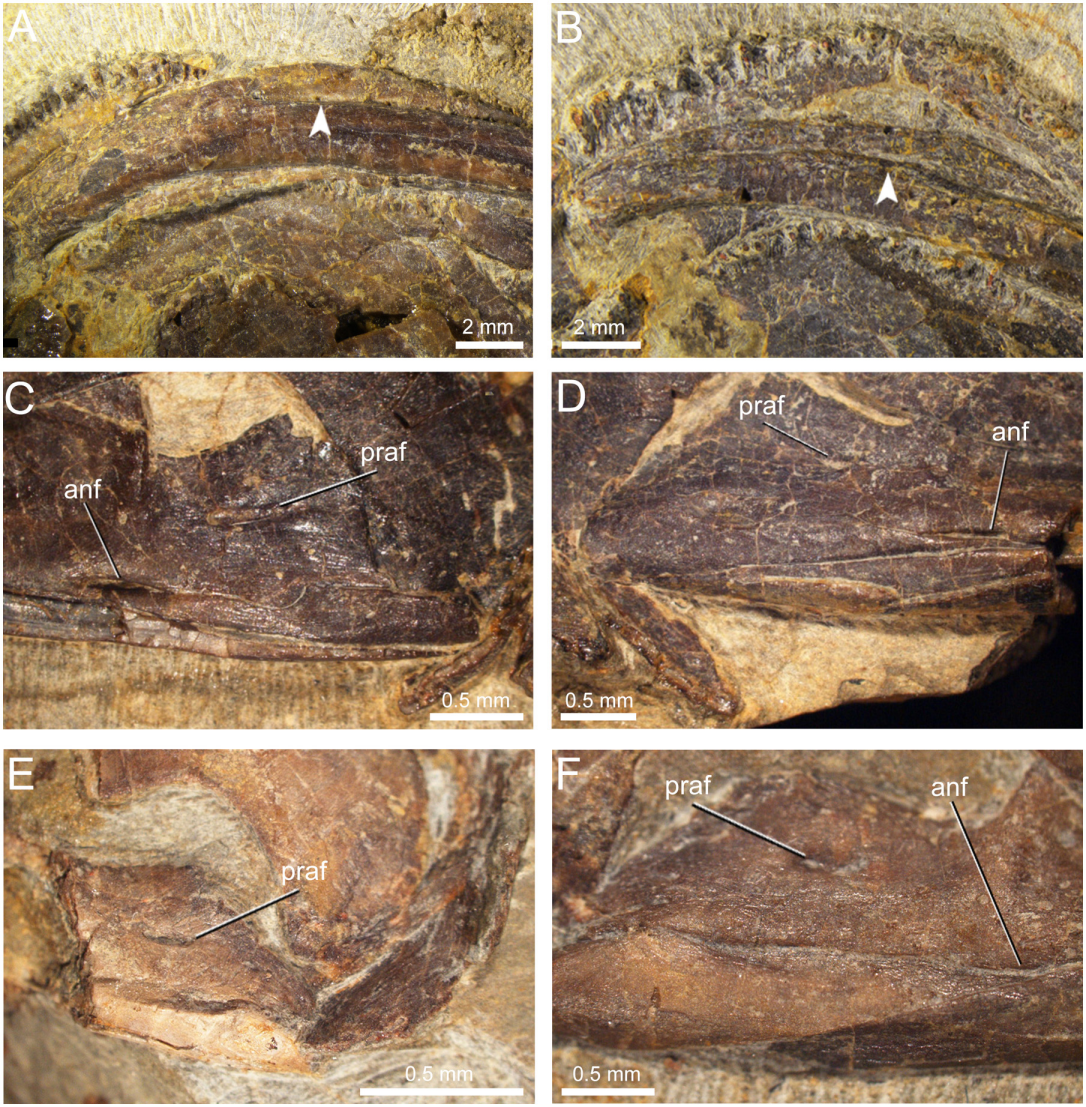
Dentary groove and angular bone in mandibles of *Qinglongtriton gangouensis*. A, lateral view of grooved left dentary in PKUP V0226; B, lateral view of grooved left dentary in PKUP V0229; note arrows in A and B pointing to dentary groove; C, D, medial view of posterior part of mandible in PKUP V0226, showing the angular fused with the prearticular but having a detectable suture on both the right (C) and left (D) mandibles; E, F, medial view of posterior part of mandibles in PKUP V0257, showing the angular remaining separate on the right (E) mandible but completely fused to the prearticular on the left (F) mandible.

**Fig 8:**
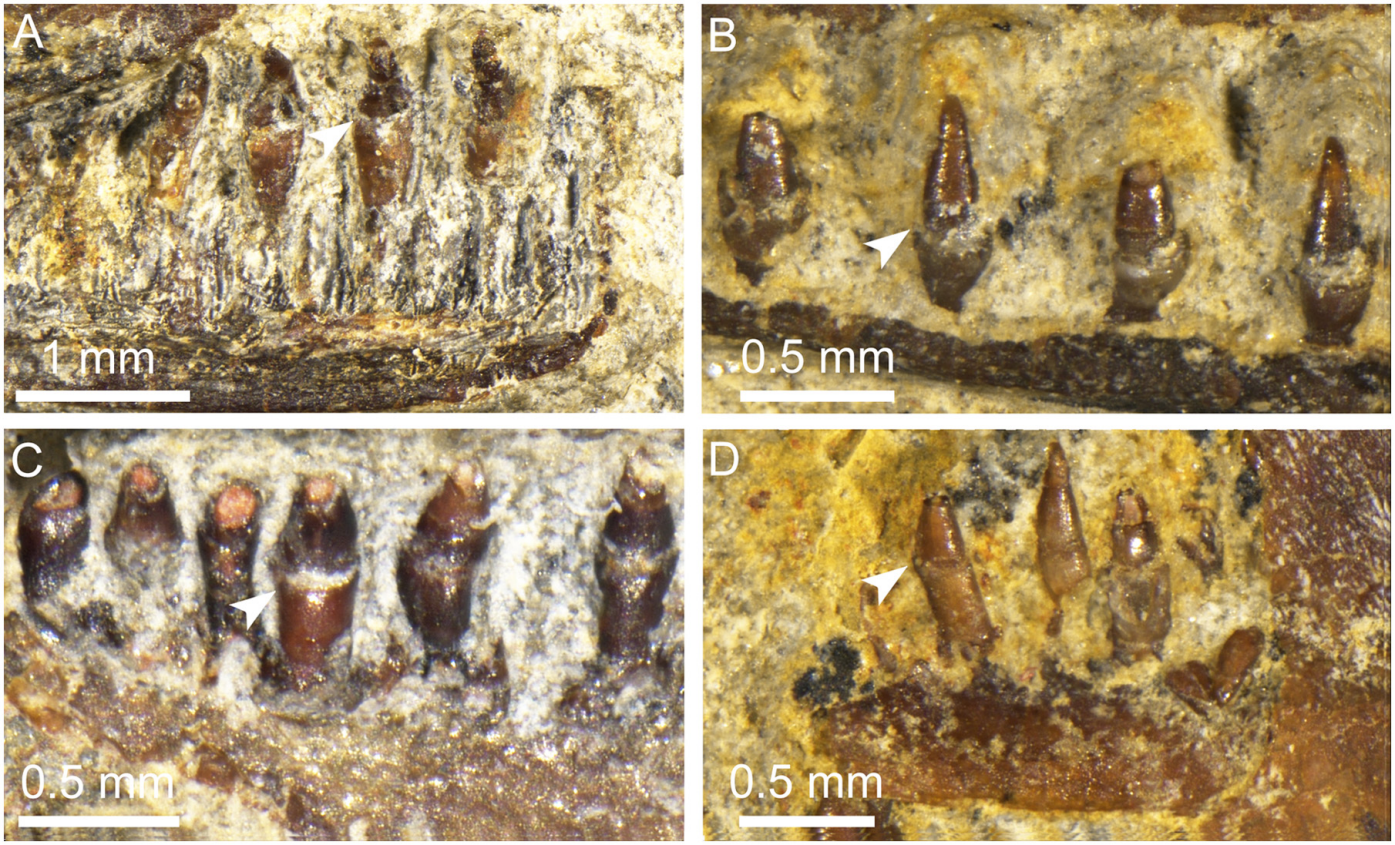
Marginal and palatal teeth of *Qinglongtriton gangouensis*. A, PKUP V0245, teeth in right premaxilla in lingual view; B, PKUP V0237, teeth in left dentary in labial view; C, PKUP V0257, teeth in right palatine in medial view; D, PKUP V0237, teeth in right coronoid in lingual view. Arrow pointing to dividing zone between basal pedicel and crown.

**Fig 9:**
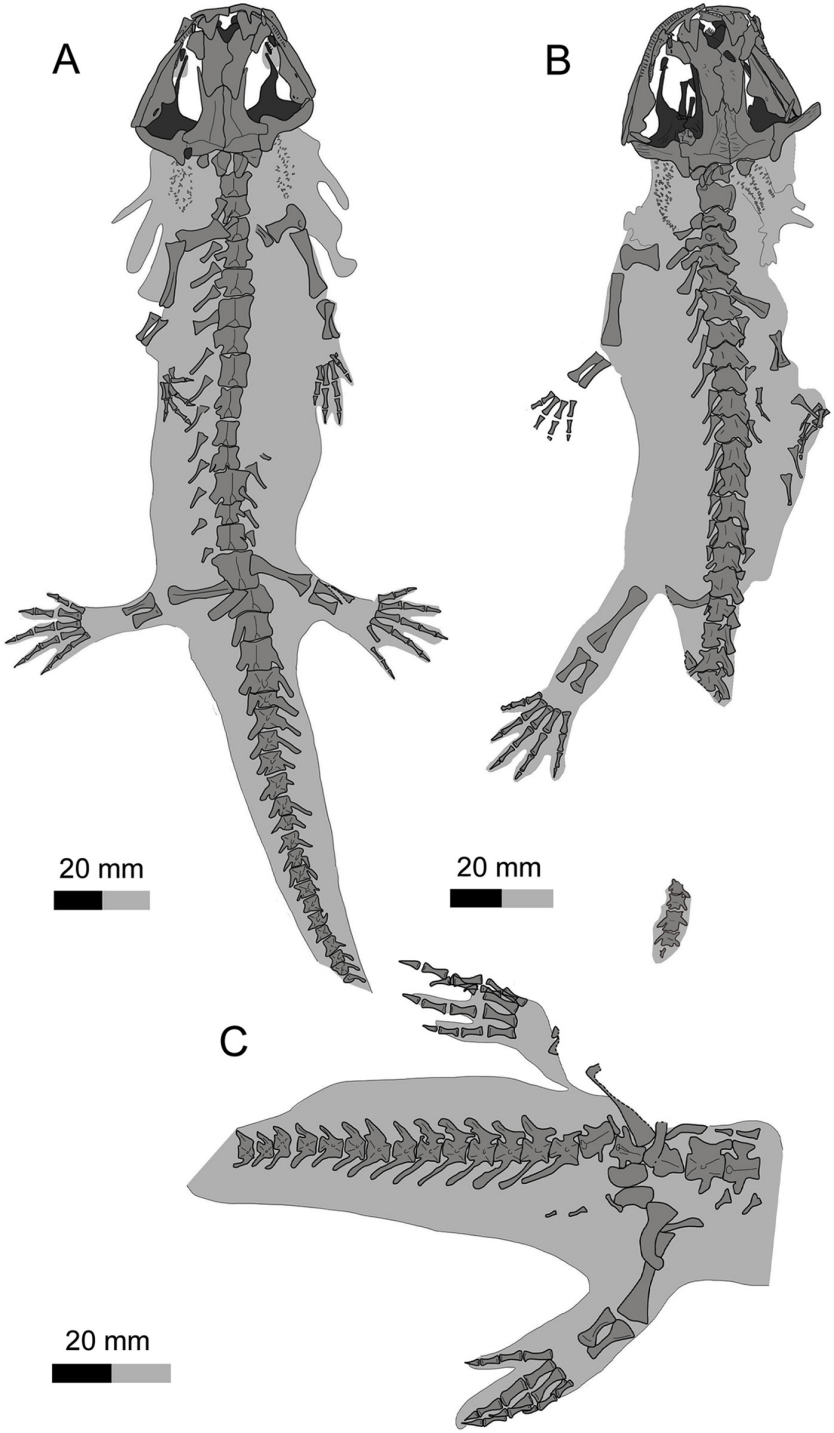
Preservation of soft-tissue impressions of *Qinglongtriton gangouensis*. A, PKUP V0241, preserving mineralized denticles of internal gill, impressions of gill filaments, body outlines of trunk, limbs and tail with skeleton, in dorsal view; B, PKUP V0237, preserving mineralized denticles of internal gill, body outlines of trunk and limbs with skeleton, in dorsal view; C, PKUP V0252, preserving soft-tissue outline of tail with caudal series rotated post-mortem to lie in right lateral view.

#### Holotype

PKUP V0226, an incomplete skeleton including articulated cranium and postcranium, with part of the tail missing (Figs 3, 5A, 6A, 7C and 7D; S1 Fig; S1 and S3 Movies).

#### Diagnosis

A basal salamandroid sharing with *Beiyanerpeton* derived features including: palatine present as discrete and dentate element; sensory groove present on external surface of premaxilla and maxilla; lateral surface of dentary deeply grooved; presacral vertebrae 15 in number. Differing from closely related *Beiyanerpeton* in having: lacrimal dorsally grooved for nasolacrimal duct; anterior ramus of pterygoid bearing teeth and directing anteromedially; ossification of orbitosphenoid absent; free operculum lacking; coronoid present as a dentate element; marginal teeth pedicellate; metacarpal II longest in manus; phalangeal formula 2-2-3-3-3 in pes. The new taxon has the basibranchial II ossified with paired anterolateral and posterolateral processes fused with a median rod as a unique feature among salamanders.

#### Etymology

“Qinglong” refers to the Qinglong Manchu Autonomous County; “triton”, suffix commonly used for salamander names; “gangou” refers to Gangou Township, in which the fossil locality occurs.

#### Referred Specimens

PKUP V0227–V0271, all specimens from the same locality and the 13^th^ layer of the stratigraphic section as the holotype.

#### Type Locality and Horizon

Fossil locality approximately 300 m northwest of Nanshimenzi village (N40°31'52*"*/E119°29'11*"*), Gangou Township, Qinglong Manchu Autonomous County, Qinhuangdao City, Hebei Province, China; the 13^th^ layer of the stratigraphic section measured as outlined in Fig 1; Upper Jurassic (Oxfordian) Tiaojishan Formation.

## Anatomical Description

### General Features

In all specimens, the snout is short and broadly rounded, although minor variations in bluntness can be seen in some specimens (Figs 3–6). Among the specimens without significant distortion, the skull is slightly longer than wide (PKUP V0226–V0229, V0233, V0235, V0237, V0238, V0240, V0242, V0253, V0254, V0256–V0258). In several other specimens, the skull is slightly wider than long, but only on account of crushing (e.g., PKUP V0230, V0231, V0236, V0241, V0245, V0250, V0251).

The new salamander is a neotenic form as clearly indicated by the presence of gill structures in the adult stage (Figs 3 and 4; S2 and S3 Figs; S1 and S2 Movies). Posterolaterally on either side of the skull the gill rakers of the internal gills are preserved as three rows of mineralized denticles, whereas the three external gill filaments are preserved as impressions. In addition, several other features in adult individuals are indicative of the neotenic nature of the new salamander (see Discussion below): a toothed palatine remains as a discrete element; the pterygoid displays a larval configuration, having a slender, toothed anterior process curving anteromedially; the coronoid is retained as a discrete and toothed element in the lower jaw; the atlas bears a pair of transverse processes; and all of the mesopodial elements are unossified.

Compared to metamorphosed salamanders, neotenic forms tend to have a large body size [53]. This is also the case with *Qinglongtriton gangouensis*, the holotype of which (PKUP V0226) has a snout-pelvic length of 155.71 mm, and a total length of ~270 mm. All other large specimens have a snout-pelvic length exceeding 100 mm. The maturity of these individuals is determined by the ossified septomaxilla, quadrate, and the hyobranchium, which are among the last cranial elements to be ossified ontogenetically [54, 55].

### Dermal Skull Roof

The premaxillae are paired, and are slightly arched anteriorly to form the bluntly rounded snout (Fig 5; S1 Fig; S3 Movie). The external surface of the premaxilla is penetrated by two or three sensory foramina for the mesial branch of the profundus nerve (CN V) as seen in extant *Salamandra* [56]. The pars dorsalis (alary process) of the premaxilla is roughly triangular, extensively overlapping the nasal, but failing to reach the frontal. The partes dorsalis of the premaxillae are medially separated by an anterodorsal fenestra. At the base of the pars dorsalis, a horizontal groove (S4 Fig) is present in association with the sensory foramina for the mesial branch of the profundus nerve (CN V) and its associated blood vessels to supply the skin of the snout [56]. The pars palatina of the premaxilla is a narrow, lingually projecting ledge connecting with the pars palatina of the maxilla to form the anterior part of the palate (e.g., PKUP V0228, V0229).

The paired nasals are broadly separated from one another by a large anterodorsal fenestra, a synapomorphy of the Salamandroidea [1, 8]. The nasal is broad anteriorly and narrowed posteriorly, forming a triangular process that overlaps the frontal. The widened part of the nasal is laterally in articulation with the lacrimal, together forming the dorsal rim of the external naris, but is separated from the maxilla by the latter element (PKUP V0230, V0237, V0241). Several tiny foramina penetrate the anterior part of the nasal (PKUP V0237, V0254), for passage of the ultimate twigs of the mesial branch of the ophthalmicus profundus nerve (CN V) as seen in extant *Salamandra* [56]. The width across the nasals is slightly greater than that of the frontals, a plesiomorphic condition in urodeles [8].

The paired frontals diverge anteriorly to form the posterior border of the anterodorsal fenestra, but meet posteriorly along the skull midline to the point where the posterior processes of the frontals diverge again to overlap the parietals. The midline suture between the two frontals curves irregularly, so that the suture is more or less sinuous rather than straight. The frontal-parietal suture is roughly “W”-shaped, resulting from the posterior process of the former element overlapping the parietal. The dorsal surface of the frontal is variably ornamented with dermal rugosities, especially in large, presumably old individuals (e.g., PKUP V0227, V0228, V0235, V0237, V0241, V0254, V0256).

The septomaxilla is small and irregular in shape, lying at the posterolateral border of the naris (Fig 5B and 5C). As seen in PKUP V0237, the tiny element is medially concave for passage of the nasolacrimal duct (see Discussion below), and it bears a pair of small arms pointing posteriorly. A septomaxilla is absent in Cryptobranchidae, Proteidae, Amphiumidae, and Sirenidae [55]; its absence in these families has been interpreted as a “paedomorphic loss” [57]. However, the absence of the septomaxilla in metamorphosed salamandrids and its presence in neotenic ambystomatids [55] are inconsistent with this interpretation.

The maxilla is reduced, being roughly the same length as the premaxilla (Fig 5). The short anterior (premaxillary) process fits in a small groove of the premaxilla, and the posterior process has a pointed tip free from contacting any bony elements. The pars facialis (dorsal process) ascends from a wide base in the anterior half of the maxilla. The dorsal border of the process is slightly notched for articulation with the lacrimal and prefrontal, but is entirely separated from the nasal by the lacrimal (PKUP V0227, V0228, V0237, V0241, V0245, V0254, V0256). The process is anteriorly notched for the external naris, and is posteriorly notched for the orbit. Several small projections are associated with these notches (PKUP V0234, V0245, V0253, V0254). Three to five foramina (S5 Fig) are scattered on the external surface of the pars facialis (PKUP V0234, V0237, V0254), and these correspond to the foramina laterale nasi for the lateral branch of the ophthalmicus profundus nerve (CN V) as seen in extant *Salamandra* [56]. At the base of the pars facialis is a horizontal groove (S5 Fig) in association with the foramina for the lateral branch of the ophthalmicus profundus nerve (CN V).

Internally, the pars palatine of the maxilla is a narrow ledge that together with the pars palatine of the premaxilla and the vomer contributes to the palate (PKUP V0228–V0230, V0237, V0241, V0245). A large foramen (occasionally two) is found at the base of the pars facialis slightly anterior to a deep fossa (PKUP V0230, V0237, V0241, V0245, V0248, V0256). This is interpreted as the infraorbital foramen, i.e., the posterior opening of the superior alveolar canal carrying a major branch of the trigeminal nerve (CN V) and its associated blood vessels. Another, smaller foramen slightly anterodorsal to the infraorbital foramen is for the lateral terminal branch of the ophthalmicus profundus of the trigeminal (CN V), as seen in extant *Salamandra* [56].

The prefrontal is wide anteriorly but is narrow posteriorly (PKUP V0227, V0228, V0230, V0233, V0237, V0241, V0245, V0254). It medially articulates with the nasal and frontal, and has a blunt posterior end that fails to contact the anterolateral process of the parietal. Anterolaterally, the widened part of the prefrontal underlies the lacrimal, and has a small part in articulation with the maxilla to close the orbital rim. Immediately above the orbital rim, the prefrontal bears a short groove (S6 Fig) that connects with the groove of the lacrimal for the passage of the nasolacrimal duct (PKUP V0228, V0230, V0237, V0245, V0254; see Discussion below).

The lacrimal is a small plate with a shallow groove (S6 Fig) on its dorsal surface (PKUP V0227, V0228, V0230, V0237, V0241, V0253, V0254, V0256, V0260). Set between the nasal and maxilla, the lacrimal enters the external naris anteriorly and overlaps the prefrontal posteriorly. The longitudinal groove on its dorsal surface carried the nasolacrimal duct, which in life drains secretions from eye glands into the nasal cavity [16, 58]. In one large specimen with a skull length of 46.18 mm (PKUP V0254), the lacrimal becomes narrow, and is scrolled into a deep trough for the nasolacrimal duct. Despite differences in shape of the bone or the depth of the groove, the dorsally grooved lacrimal is a unique feature of the new salamander. In extant salamanders, a lacrimal is found in hynobiids, dicamptodontids, and rhyacotritonids, but in these species the nasolacrimal duct passes through the lacrimal ventrally ([59]; see Discussion below).

The paired parietals contact one another along a straight suture at the midline. Anteriorly, the parietals are extensively overlapped by the frontals. A large part of the lateral border of the parietal curves downward as a well-developed descending flange, because an ossified orbitosphenoid is absent (see below). A slender anterolateral process of the parietal extends along the lateral margin of the frontal, approaching but failing to contact the prefrontal; thus, the parietal contributes to most of the medial border of the orbit (PKUP V0227–V0229, V0235, V0237, V0241, V0254). The parietal also bears a short, boot-like posterolateral process laterally in contact with the squamosal (PKUP V0227, V0229, V0230, V0233, V0235, V0237, V0241, V0245, V0246, V0250, V0253, V0254). In those large specimens (e.g., PKUP V0235, V0245, V0254), the parietal table is heavily ornamented with vermiculate rugosities along the midline suture, whereas in those smaller specimens (e.g., PKUP V0228, V0257, V0258) ornamentation is much less pronounced. The posterior border of the parietal table forms a transverse bony ridge, from which the parietal turns downward to form a flange that together with the opisthotic and exoccipital closes the braincase posteriorly. The external concave surface of the flange is the cervical epaxial fossa for attachment of the intervertebral epaxial muscles as seen in extant *Amphiuma* [60].

### Suspensorium

The squamosal is roughly “T”-shaped, with an expanded proximal end and a blunt distal end (Figs 4 and 5). The proximal end of the squamosal is in broad contact with the posterolateral process of the parietal. Presence of a parietal-squamosal contact is a plesiomorphic condition at the level of Caudata, as known from the stem caudates *Karaurus* and *Kokartus* [61, 62]. Generally, the squamosal is dorsally convex and ventrally concave. The dorsal surface bears a prominent ridge running from the proximal to the distal end along the anterior margin. This ridge marks the attachment of the adductor mandibulae externus as known from extant salamanders [63]. Ventrally, the squamosal overlaps the pterygoid and quadrate (Figs 4 and 5; S1 Fig; S3 Movie).

The quadrate is small but well ossified as a principal element of the suspensorium. Several specimens (e.g., PKUP V0231, V0234, V0241, V0245, V0248, V0255, V0257, V0260) with the quadrate preserved in life position show that it is transversely oriented, whereas in other specimens (e.g., PKUP V0226, V0228, V0229, V0236, V0239, V0240, V0242, V0246, V0247, V0249, V0252, V0253, V0256, V0258, V0259) the bone is disarticulated and slightly rotated to an oblique or even a perpendicular position in relation to the squamosal, clearly an artifact of preservation. The quadrate has a long ascending process extending about half the length of the anteroventral border of the squamosal. At the base of this process, the quadrate articulates with the squamosal by clasping its anterodistal end, as evidenced by an articular facet on the squamosal in those specimens in which the quadrate has been disarticulated from the squamosal. The distal end of the quadrate is slightly expanded, and is ventrally convex forming a condyle for articulation with the mandible (PKUP V0227, V0230, V0235, V0237, V0241, V0245, V0254). The expanded distal end also meets the pterygoid at the craniomandibular joint. A small quadrate foramen is identified in several specimens (PKUP V0226, V0231, V0234, V0237, V0241, V0245, V0254) penetrating the expanded distal part of the element (Fig 5A and 5C). This is the opening for the passage of the branch of the posterior condylar artery and vein as generally seen in other tetrapods [64].

The pterygoid is triradiate, but displays a larval configuration (Fig 6). That is, the anterior palatal process is markedly elongated and curved anteromedially. However, the process is free from contacting any bony element including the palatine (PKUP V0226–V0229, V0235–V0238, V0240, V0241, V0245, V0246, V0248, V0249). The process ventrally bears a single tooth row along its anterior portion. The medial process is extremely short, and it bears a small facet for articulation with the ala of the parasphenoid. The quadrate process is expanded posterolaterally, and distally articulates with the quadrate and the squamosal. In several specimens (PKUP V0226, V0237, V0240, V0254), the left pterygoid is penetrated by a small foramen behind the tooth row. Such a foramen is probably the result of natural bone resorption, as commonly seen in extant and extinct salamander species, such as *Paradactylodon persicus* (Hynobiidae), *Onychodactylus japonicus* (Hynobiidae), *Ambystoma gracile* (Ambystomatidae), *Triturus karelinii* (Salamandridae) and *Dicamptodon ensatus* (Dicamptodontidae) [65, 66].

### Palate

The palate consists mainly of the paired vomers and palatines, and the partes palatina of the premaxillae and maxillae. In addition, the broad, anterior part of the parasphenoid contributes to the formation of the palate. The vomers are strip-like, obliquely arranged, and are separated at the skull midline by the anteromedial fenestra (Figs 3 and 6). The vomers articulate with the partes palatina of the premaxilla and maxilla anterolaterally, and in palatal view overlap the parasphenoid posteromedially (PKUP V0226, V0234, V0237). Each vomer bears a curved tooth row, paralleling the maxillary arcade. Ventrally, the vomer carries two short grooves behind the tooth row in all specimens having this part exposed (PKUP V0226, V0229, V0235, V0246). One groove is perpendicular to, and the other lying posteromedial to, the vomerine tooth row (Fig 6A). These were probably for nerves and blood vessels associated with the palate. Close to the end of the vomerine tooth row, the posterolateral border of the vomer is indented an incipient notch for the choana (Figs 3 and 6).

The palatine is a small, almond-shaped plate, with its pointed end oriented posteriorly. It lies close to the anterior process of the pterygoid, but clearly remains separate from the latter element, although displaced in several specimens overlapping either the vomer or pterygoid. As observed in PKUP V0257, the palatine bears a straight tooth row along its lateral margin. Besides the basal salamandroid *Beiyanerpeton*, *Qinglongtriton* is the second Jurassic salamander known as having a discrete and toothed palatine in the adult stage. The accumulating evidence supports the argument by Gao and Shubin [8] that the presence of a separate palatine as a tooth-bearing element in the palate is probably a plesiomorphic condition for Salamandroidea.

The parasphenoid is a large, azygous bony plate that forms the braincase floor and part of the palate. The cultriform process is narrow at its base, but widens abruptly towards its anterior end, where it underlies the vomers anterolaterally in palatal view. The anterior margin of the process is notched for the posterior border of the anteromedial fenestra (Figs 3 and 6). The posterolateral ala is short, triangular, and is laterally in contact with the medial process of the pterygoid. As observed in several specimens (PKUP V0226, V0246, V0257, V0267), the alae are grooved for the passage of the internal carotid arteries, with the internal carotid foramen opening within the groove.

### Braincase

The large and azygous parasphenoid forms the braincase floor and part of the palate as has been described above. No orbitosphenoid (sphenethmoid) has been observed in dorsal or palatal view of the available specimens. CT scans of several large specimens confirm that paired orbitosphenoids are completely lacking (Fig 6; S1 Fig; S3 Movie). Among extant salamanders, proteids are the only group that lacks a free orbitosphenoid [55, 67]. However, the orbito-temporal region in proteids is laterally covered by a bony wall, which has no suture can be delimited from the parietal but is penetrated by the foramen opticum and foramen oculomotorium as commonly seen in other salamanders; this is obviously not the case in *Qinglongtriton*. Because there is no trace of an orbitosphenoid even in adult specimens, the element can be interpreted as cartilaginous in the new salamander.

The prootic, opisthotic and exoccipital are three separately ossified elements. The prootic is a small plate that is irregular in shape, concave internally and convex externally. It articulates with the parietal dorsally and with the parasphenoid ventrally. The prootic is posteriorly notched for the fenestra ovalis, which is covered by the footplate of the stapes (Fig 6).

The opisthotic is a medially concave bony plate. Ventrally, it articulates with the posterior margin of the parasphenoid and the footplate of the stapes, and dorsally with the posterior edge of the parietal. Medially, the opisthotic meets with the exoccipital along a sutural line.

The exoccipitals are paired elements that meet both dorsally and ventrally to encircle the foramen magnum. Anteriorly, the exoccipital articulates with the parietal and parasphenoid. The ventrolateral surface of the exoccipital is perforated by a foramen, the postotic foramen, for the passage of CN XII [56]. Posteriorly, each exoccipital bears an occipital condyle, articulating with the corresponding atlantal cotyle of the atlas (PKUP V0226–V0228, V0234, V0237, V0246; see below).

The stapes (= columella) is observed in several specimens (PKUP V0226–V0229, V0246, V0249, V0254, V0257) as having a thickened and rounded footplate covering the fenestra ovalis. The footplate is fused with a short and stout stylus. The stapes is perforated by a stapedial foramen (S1 Fig; S3 Movie) at the base of the stylus for the passage of the stapedial artery [68] and/or the hyomandibular trunk of the facial nerve (CN VII). This probably is a plesiomorphic condition, clearly different from all extant salamanders (see Discussion below). No separate operculum is ossified in *Qinglongtriton*.

### Mandible

The mandible consists of the dentary, coronoid, and prearticular. In addition, the angular, developed as a small posteroventral splint, is at least partly fused to the prearticular (see below). The mentomeckelian is synostotically united with the anterior end of the dentary and bears a tiny posterior mental process at the mandibular symphysis (Fig 6).

The dentary is the principal tooth-bearing element, covering most of the lateral aspect of the lower jaw. Four or five mental foramina can be seen in a row along the anterior lateral surface of the dentary. These foramina are probably for the passage of the mandibular branch of the trigeminal and facial nerves (CN V and VII) and their associated blood vessels for nourishing the lower lip [56]. In close association with the row of foramina, a deep groove is consistently observed in different specimens (e.g., PKUP V0226, V0234, V0238, V0249) on the labial side of the dentary (Fig 7A and 7B; S1 Fig; S3 Movie). Although undescribed in the original publication by Gao and Shubin [8], several newly prepared specimens revealed that such a distinct groove is also present in *Beiyanerpeton*. Based on our knowledge of salamander anatomy [60, 69–71], the deep groove in labial surface of dentary is probably for passage of the external branch of the ramus mandibularis nerve (CN V) and its associated blood vessels. A similar groove, but present in a much less extent, has been described in amphiumids and sirenids [69–71]. The groove extends along the posterior half of the dentary in the extant salamanders as cited above, but it extends further anteriorly close to the mandibular symphysis in both *Beiyanerpeton* and the new salamander described here.

The coronoid is a toothed element, narrow and elongate, attached to the anterodorsal part of the prearticular (Fig 5A and 5C; S1 Fig; S3 Movie). It bears a single tooth row along its dorsal margin parallel to the dentary tooth row. The coronoid tooth row terminates anteriorly at a point slightly anterior to the middle level of the dentary tooth row and posteriorly at the same position as the end of the dentary tooth row (PKUP V0237, V0254). A coronoid is absent in most salamanders, but is present in sirenids and proteids as a toothed element, and in dicamptodontids as a toothless element [55].

The prearticular as best shown in several specimens (PKUP V0230, V0237, V0254) is a large and wedge-shaped plate attached to the medial side of the dentary. It tapers anteriorly to a point posteroventral to the coronoid; thus, the prearticular is far from reaching the jaw symphysis. The posterior part of the prearticular deepens significantly, and expands dorsally into a coronoid process for insertion of a portion of the mandibular adductor muscles. A small, circular prearticular foramen (inferior dental foramen of [56]) opens posteroventral to the coronoid process (Fig 7C–7F). This foramen is probably for the passage of the inferior alveolar ramus of the facial nerve (CN VII) and the alveolar artery as in extant *Salamandra* [56].

The angular is a small element that displays various degrees of fusion with the prearticular in different specimens. In the largest individual in the collection (PKUP V0254), with a skull length of 46.18 mm, the angular is fully fused to the prearticular without any trace of sutural contact. In another large specimen (holotype PKUP V0226), with a skull length of 45.11 mm, the angular is fused to the prearticular but still has a detectable suture (Fig 7C and 7D); whereas in two other, smaller specimens (PKUP V0237, V0257), with skull lengths of 35–36 mm, the angular is either fused to or remains partly separate from the prearticular. PKUP V0257 even displays asymmetrical fusion of the element, with the angular fused to the prearticular in the left mandible but separate in the right mandible (Fig 7E and 7F). Comparison of these specimens indicates that the fusion of the angular to the prearticular happened fairly late in ontogeny. As observed in the holotype and several other specimens, a large angular foramen opens anteroventrally to the prearticular foramen at the level of where the prearticular-angular suture is supposed to occur (Fig 7C, 7D and 7F). The foramen is dorsoventrally narrow and elongate, with its opening directed medially.

No articular has been discovered in any available specimens, probably due to its cartilaginous nature or fusion with the prearticular, as occurs in different families in both Cryptobranchoidea and Salamandroidea [72]. Based on our knowledge of extant salamanders, lack of the articular is not strictly related to neoteny, because the bone is present in both neotenic (cryptobranchids and sirenids) and metamorphosed (e.g., hynobiids, salamandrids, dicamptodontids) forms, and is also absent in both neotenic (proteids and amphiumids) and metamorphosed (e.g., plethodontids) forms (see [55] for details and citations).

### Hyobranchial Apparatus

The ossified hyobranchial apparatus consists of the paired hypobranchial I and II, and a median basibranchial II (os thyroideum or os triangulare of some authors [55, 56]). No ceratobranchials are ossified in the specimens at hand, although the presence of rows of gill rakers indicates that the ceratobranchials were almost certainly present as cartilaginous structures in life.

Hypobranchial I is a pair of straight rods, lying obliquely anterolateral to hypobranchial II (Figs 3 and 6; S1 Fig; S3 Movie). The paired rods ventrally bear a short and shallow groove for the passage of the hypobranchial artery [73]. Hypobranchial II is another pair of rods that are in similar length and robustness as hypobranchial I, but are slightly curved posterolaterally. Hypobranchial II also bears a shallow groove on the ventral surface for the passage of hyobranchial arteries [73].

Basibranchial II is a median element, displaying a shape that differs from this structure in all known salamanders in the adult stage (Figs 3 and 6; see Discussion below). The anterior part of the basibranchial II is basically anchor-shaped, as the median rod is fused with a pair of anterolateral processes. The process is flat, and medially has a thin crest presumably for attachment of the M. geniohyoideus and M. rectus cervicis superficialis as in extant salamanders [56, 74]. The median rod extends posteriorly as a spike-like process, and is fused with a pair of posterolateral processes immediately posterior to the anchor-shaped portion (Figs 3 and 6). In juvenile specimens (e.g., PKUP V0257, V0267), however, the posterolateral processes are lacking; therefore, ontogenetic change of the basibranchial II is documented by ossification of an extra pair of posterolateral processes in adults (see Discussion below).

### Dentition

Tooth-bearing elements in *Qinglongtriton* include the premaxilla and maxillae in the maxillary arch; vomers, palatines and pterygoids in the palate; and the dentary and coronoid in the lower jaw. Each of these bones bears a single tooth row (monostichous), and all teeth are monocuspid with a pointed tip (Fig 8).

As best observed from two specimens (PKUP V0237, V0254), marginal teeth are pedicellate, having the basal pedicel and crown separated by a poorly mineralized dividing zone (Fig 8). The teeth in the premaxilla and maxilla consistently number as 18–20 on each of these elements in large specimens (PKUP V0226, V0229, V0234, V0237), the same number is also seen in a juvenile specimen (PKUP V0264: SKL = 18.28 mm, SPL = 64.56 mm), although the dentary tooth row is not exposed in the latter specimen. The dentary tooth row has ~50 tooth positions in two specimens (PKUP V0237, V0254). A complete coronoid tooth row is observed in PKUP V0254, which bears ~20 pedicellate teeth.

Palatal teeth are pedicellate, but sub-pedicellate teeth with a lingually globular/fibrous ring-like region can be occasionally observed as a developmental feature. Vomerine teeth are best exposed in PKUP V0229, are about 23 in number, and are arranged in a single, curved row. A similar number is also revealed in a CT-scan of other two specimens (PKUP V0227, V0228). There are 10 or 11 teeth in the palatine (PKUP V0237, V0257), and ~22 in the pterygoid (PKUP V0226).

### Axial Skeleton

The vertebral column of *Qinglongtriton* consists of 15 presacrals, including the atlas, 14 trunk vertebrae, one sacral, three caudosacrals, and as many as 33 caudals (Figs 3 and 4; S1 and S2 Movies). The vertebral centrum is amphicoelous as in many other salamanders, except salamandrids and plethodontids and extinct batrachosauroidids that have opisthocoelous type of centrum [61, 75]. The number of the presacrals is invariably constant, whereas the number of caudals varies among different specimens. The tail is shorter than the snout-pelvic length in adult (PKUP V0233) and large juvenile individuals (PKUP V0236, V0266), but vice versa in small juveniles (e.g., PKUP V0263).

The atlas is hourglass-shaped, and is slightly wider and longer than the trunk vertebrae as in most other salamanders. The atlas bears a short, triangular odontoid process (tuberculum interglenoideum) anteroventrally. The paired cotyles are cup-like facets for articulation with the occipital condyles. The prominent crest extends along the entire length of the neural arch, and slightly over the first trunk vertebra. In ventral view, the atlas lacks a subcentral keel, but has a large median depression immediately behind the odontoid process (Figs 3 and 6). A similar median depression of the atlas is known from the stem caudate *Urupia* [76] and some but not all extant salamanders (see Discussion below).

In lateral view of the atlas, a spinal nerve foramen opens on each side at the base of the neural arch. Interestingly, just below the spinal nerve foramen the atlas bears a small projection, which corresponds to the “rudimentary transverse process” of Mivart [77] for a similar structure in proteids and sirenids, followed by Gilbert [78] for *Necturus*, Skutschas and Martin [62] for the stem caudate *Kokartus*, and Skutschas and Krasnolutskii [76] for *Urupia*. The centrum also displays a short ridge laterally, proceeding from the posterior edge to the transverse process (PKUP V0226, V0249, V0256).

In the trunk region, the vertebral centrum tends to increase in length from the second to the 11th or the 12th vertebra, and then becomes shorter towards the sacral vertebra. As seen in the holotype (PKUP V0226), the shortest vertebra (first trunk) is only half the length of the longest one (11th trunk). In dorsal view, the neural arch dorsally carries a low crest that runs along much of the length of the vertebra and continues onto the neural spine, which rises posterodorsally as a short bar that is triangular in cross-section. The dorsal tip of the spine ends in a facet, probably for attachment of a tendon of the M. dorsalis trunci [56]. Spinal nerve foramina penetrate the neural arch anterior to the transverse process for the first two trunk vertebrae, but posterior to the transverse process for the rest of the trunk series (PKUP V0228, V0229, V0231, V0251, V0256). The anterior two trunk vertebrae each ventrally lack a subcentral keel, but bear a depression with that of the second trunk often deeper than the first. The remaining trunk vertebrae lack a ventral depression, but bear a subcentral keel with a foramen opening on each side of the keel. The subcentral keel extends the length of the centrum in the anterior vertebrae, and then gradually shortens to become limited to the middle portion of the centrum in the posterior portion of the trunk series. Laterally, all trunk vertebrae bear a pair of rod-like transverse processes. The transverse process is weakly bifurcated, with two heads closely appressed with each other. The process is half the length of the centrum, is directed posterolaterally, and bears two facets distally for articulation with the bicapitate ribs.

The first three pairs of ribs are obviously more robust than the remaining ones, having an expanded distal end for attachment of the pectoral muscles. In PKUP V0226 and V0258, a short uncinate process is present on the first or the third trunk rib, for attachment of epaxial muscles. Starting from the fourth pair, the trunk ribs tend to become shorter posteriorly, with the last pair being merely triangular stubs. The anterior ribs are bicapitate, but have the tuberculum and capitulum connected by an extremely thin bony web. Starting from the fourth pair, the remaining ribs have a small notch developed between the two heads; in some cases the dorsal tuberculum is more prominent than the ventral capitulum (PKUP V0226, V0237, V0240, V0241). The possession of bicapitate trunk ribs gives a clear indication of membership of *Qinglongtriton* in the suborder Salamandroidea.

The sacral vertebra is the same in length as the last trunk vertebra. Dorsally, the neural spine is as prominent as in the trunk region. Ventrally, the subcentral keel is limited to the middle portion of the centrum, with a subcentral foramen on each side of the keel. Laterally, the transverse processes are stouter and longer than those of the trunk vertebrae. A spinal nerve foramen opens posterior to the transverse process. The sacral rib is long and curved, about the same length as the ilium (Figs 3 and 4).

Several rib-bearing vertebrae immediately following the sacral are often referred to as the caudosacrals [79]. In *Qinglongtriton*, three pertain to in this series: preserved in a dorsoventral position, they clearly bear free ribs (Figs 3 and 4). The neural spine is limited to the posterior part of the neural arch (PKUP V0228, V0231, V0238). Ventrally, the subcentral keel and foramina on the three caudosacrals are unclear in the holotype, but a CT-scan of PKUP V0228 reveals the same pattern as in the sacral and posterior trunk vertebrae. The transverse process in these caudosacrals is about half the length of the centrum, and is directed posterolaterally. The free ribs are slender and slightly curved, and have the same length as their corresponding transverse process.

Beginning from the first caudal vertebra, the tail commonly has been rotated taphonomically at a right angle to the horizontal body plan. The neural and haemal arches are all well ossified, and the haemal spine of the first caudal vertebra is bent at a greater angle than those of the other caudals. Spinal nerve foramina penetrate the neural arch posterior to the transverse process of the caudal vertebrae (PKUP V0231). The number of ossified caudal vertebrae increases ontogenetically, as small individuals possess fewer caudals (25 in PKUP V0263; 24 in PKUP V0266) than large individuals (>29 in PKUP V0226, 30 in PKUP V0233 and >27 in PKUP V0236).

### Appendicular Skeleton

The ossified pectoral girdle consists of a pair of scapulocoracoids (Figs 3 and 4; S1 and S2 Movies), as in other salamanders except for sirenids that have a separate coracoid [16, 57]. The scapular portion is trapezoidal in shape, having the expanded distal end about twice the width of the proximal constriction where it fuses with the coracoid plate. The coracoid portion is expanded into a fan-shaped structure, which in lateral view is convex anteriorly and concave posteriorly.

The glenoid fossa, an ovoid socket, is developed within a posterior concavity of the coracoid plate. The supracoracoid foramen (coracoid foramen) penetrates the thickened coracoid plate anterior to the glenoid fossa as can be seen in both the lateral and medial side of the plate. The foramen in life is for the passage of the supracoracoideus nerve and the corresponding artery and vein [56]. No suprascapula, procoracoid, or sternum is preserved; these elements are all cartilaginous in extant salamanders and presumably were in *Qinglongtriton*.

The humerus is straight, having a short shaft and slightly expanded proximal and distal ends (S7A Fig). Both the proximal and distal ends are slightly concave, implying where were capped with a cartilaginous humeral head and distal condyles, respectively. On the flexor side, the crista ventralis is merely a short smooth ridge running along the pre-axial border from the proximal end to the shaft, and lacks any recognizable projection. Opposite to the crista ventralis and on the extensor side, the proximal end bears a small tubercle connected with a small ridge as the crista dorsalis, presumably for insertion of M. subscapularis [56]. Distally, both the olecranon fossa on the extensor, and the fossa cubitalis ventralis on the flexor side are recognizable. The radial and ulnar condyles are not ossified.

The radius is roughly the same in length as the ulna or is slightly shorter than the latter. The radius is slightly wider distally than proximally, whereas the ulna exhibits the converse, with its proximal end wider than the distal end (S7A Fig). The ulna proximally bears a blunt olecranon process, anterodorsal to which is the articular facet for the humeral condyle.

No mesopodial elements are preserved in any of the specimens in the collection, and this lack of ossification of the mesopodium is obviously a neotenic feature of *Qinglongtriton*. The metacarpals are all dumbbell-shaped, but are slightly different in length and robustness. Metacarpal II is the longest and most robust in all available specimens; metacarpal I and IV are subequal in length and are the shortest.

The phalanges are also dumbbell-shaped, except the terminal phalanges, which have a pointed tip. In all specimens, the third digit is the longest and the first the shortest. The phalangeal formula is 2-2-3-2, a plesiomorphic pattern for salamanders [80].

The pelvic girdle consists of paired ilia and ischia, whereas the pubis and ypsiloid are not preserved and are assumed to be cartilaginous as in extant taxa [57]. The ilium resembles a curved club, having its dorsal end slightly expanded for the attachment of the M. extensor iliotibialis [56]. The ventral end is expanded to form part of the acetabulum. In PKUP V0253, the acetabulum is a shallow fossa irregular in shape. The ischium is a plate, having a slightly concave lateral border and a straight medial edge for the symphysis (Figs 3 and 4).

The femur is straight, with a short shaft and expanded proximal and distal ends (S7B Fig). The femoral head and distal condyles were probably cartilaginous, as indicated by concave surfaces on both ends of the shaft. The femur is slightly longer than the humerus, and is about twice the length of the tibia or fibula. The femur clearly lacks a hooked trochanter, with this structure merely a tiny process projecting anteroventrally, connected with the trochanter crest as a thin ridge along the proximal one-third of the femur. The trochanter groove is a deep fossa directly posterior to the anterior trochanter crest. The distal end of the femur bears a shallow trochlear groove, separating the relatively larger tibia condyle from the smaller fibular condyle as seen in several specimens (PKUP V0236, V0248, V0253).

The tibia and the fibula are both straight. The tibia is slightly wider proximally than distally, whereas the fibula is wider distally than proximally (S7B Fig). The tibial crest is a short triangular ridge for insertion of the tendon of M. extensor iliotibialis [56]. The fibula is slightly shorter than the tibia.

No tarsals are ossified. The metatarsals are all dumbbell-shaped, with metatarsal III consistently the longest, and metatarsal I the shortest. Metatarsal II and IV are subequal in length, and metatarsal V is the second shortest.

All phalanges are more or less dumbbell-shaped, except the terminal phalanges, which are slenderly pointed. The third digit is the longest and the first the shortest, corresponding to the relative length of the metatarsals. The phalangeal formula is 2-2-3-3-3 in all specimens but PKUP V0256, in which it is 2-2-3-4-3.

### Soft Tissues

Many specimens are preserved with soft-tissue impressions of the eye, body outline, and external gill filaments (Figs 3, 4 and 9; S2 and S3 Figs). Mineralized denticles commonly termed gill rakers are not classified as soft tissues but are part of the internal gills and, hence, are described here.

All specimens are preserved with gill rakers, and many with gill filaments as evidence of external gills. Gill rakers are tiny triangular denticles arranged in three rows, with the paired denticles preserved in interlocked position like the teeth of a zipper. As in extant salamanders [74], the gill rakers were used for supporting the internal gills and as strainers in suction feeding. The external gills are preserved as impressions of gill filaments, three in number on each side.

Eyes are preserved as rounded dark stains within the orbit (PKUP V0227). Scleral cartilages might be expected to be present as typically seen in other neotenic salamanders, but left no trace in these specimens.

Body outlines of the trunk, tail and appendages are preserved in many specimens (e.g., PKUP V0228, V0240, V0241, V0244, V0245, V0252, V0253). The trunk region is stout and the limbs short and robust (Fig 9A and 9B; S2 and S3 Figs). A few relatively slim-bodied specimens with a proportionally longer tail are probably male individuals as in extant salamanders [81]. Those specimens (PKUP V0241, V0245, V0252) show that the tail is deep and laterally compressed, having a low dorsal fin and a deep ventral fin (Fig 9C). The laterally compressed tail and well-developed tail fin are consistent with the supposed aquatic life style of *Qinglongtriton*, as evidenced by its neotenic features.

## Results and Discussion

Compared to other known fossil and extant salamanders, the new taxon has several significant features that may shed light on character evolution in salamanders. We discuss these characters as below.

### Significance of *Qinglongtriton* for Character Distribution and Evolution in Salamanders

#### Lacrimal and Nasolacrimal Duct

**Fig 10:**
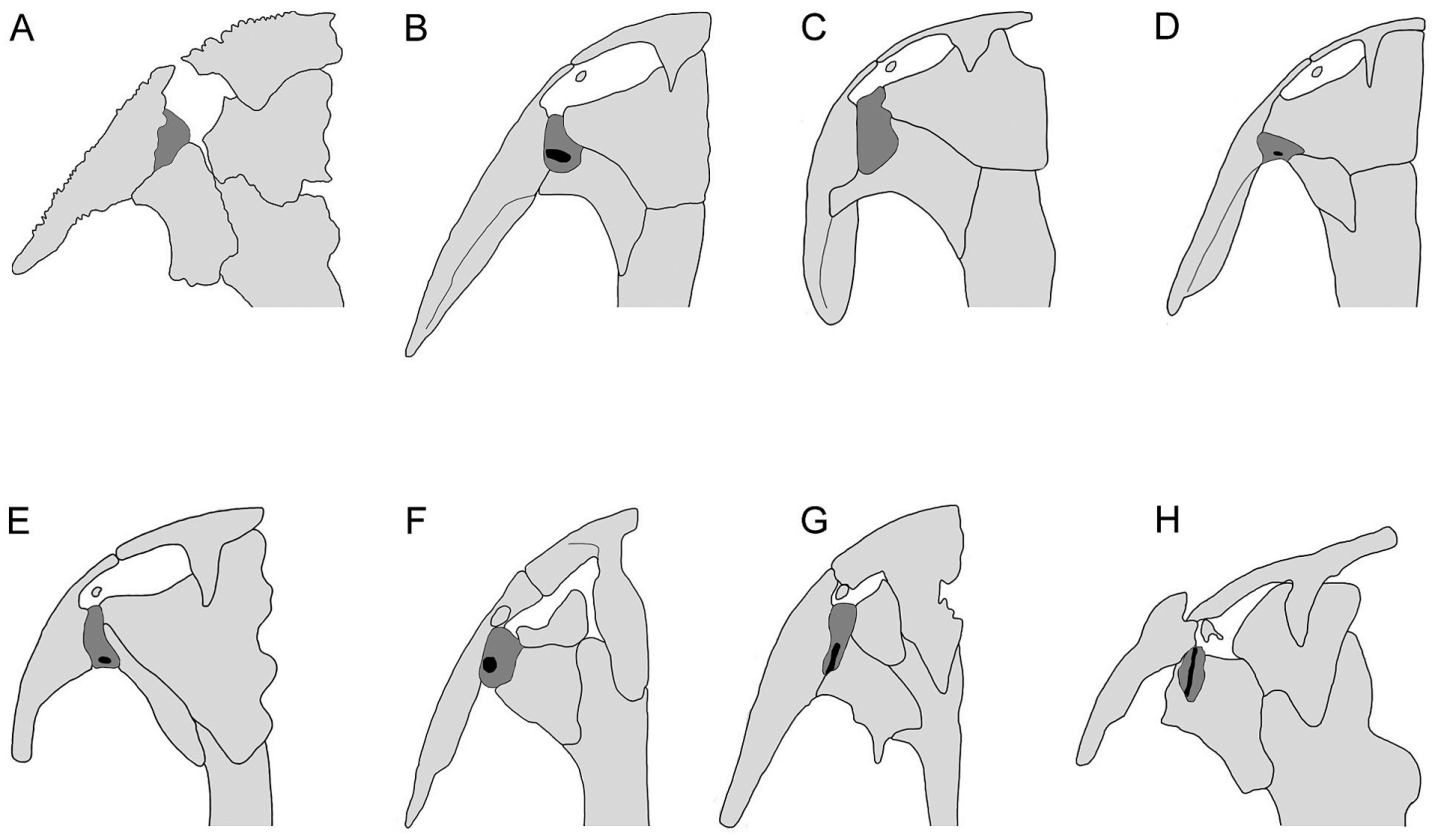
Comparison of position of lacrimal in relation to external naris and orbit in different groups of salamanders. A, *Karaurus* (Karauridae); B, *Salamandrella* (Hynobiidae); C, *Batrachuperus* (Hynobiidae); D, *Hynobius* (Hynobiidae); E, *Ranodon* (Hynobiidae); F, *Rhyacotriton* (Rhyacotritonidae); G, *Dicamptodon* (Dicamptodontidae); H, *Qinglongtriton* based on PKUP V0237. A, based on Estes [61]; B–E, based on Fei et al. [82]; F, based on AmphibiaTree [83]; and G, based on Wake [66]. All are depicted in dorsal view, lacrimal is shaded in dark gray and opening of nasolacrimal duct is shaded in dark. Not in scale.

A lacrimal is present in extant hynobiids, dicamptodontids, and rhyacotritonids, whereas other salamanders lack this element [55, 57]. Because the stem caudate *Karaurus* also has a lacrimal, the presence of this element in extant salamanders is probably a plesiomorphic feature. In those salamanders in which the lacrimal is present at the adult stage, it is a small plate between the maxilla and the nasal/prefrontal (Fig 10). The position of the lacrimal relative to the narial opening or to the orbit is an informative feature in the classification of hynobiids [82]. Within the Hynobiidae, the lacrimal variably enters the naris, the orbit or both. The lacrimal enters only the naris in the Dicamptodontidae, whereas it enters both the naris and orbit in the Rhyacotritonidae. Taking the stem caudate *Karaurus* for out-group comparison, it seems that the lacrimal entering only the naris is a plesiomorphic condition for salamanders.

*Qinglongtriton* displays the inferred plesiomorphic condition of the lacrimal, with a small lacrimal entering only the naris (PKUP V0227, V0228, V0237, V0241, V0253, V0254, V0256, V0260). However, the new salamander uniquely has an open groove on the dorsal surface of the lacrimal (S6 Fig), different from all other lacrimal-bearing salamanders. The groove is for the passage of the nasolacrimal duct, which is a soft-tissue tube to conduct secretions from the eye gland into the nasal cavity [16, 58]. In those salamanders having a lacrimal bone (hynobiids, dicamptodontids, and rhyacotritonids), the nasolacrimal duct normally enters a small foramen posterodorsally on the lacrimal bone, passes through the lacrimal canal, and then exits from another foramen on the anteroventral edge of the bone. This pattern is seen in *Hynobius*, *Ranodon*, and *Onychodactylus* [73, 84], and in *Dicamptodon* and *Rhyacotriton* [66, 83].

Developmental evidence provides the clue for the dorsally open condition of the lacrimal canal in *Qinglongtriton*. As known from extant salamanders (e.g., *Ranodon* and *Onychodactylus*), the lacrimal first occurs as a thin plate at or before metamorphosis, after the formation of the nasolacrimal duct; then the thin plate grows to enclose the canal at or soon after metamorphosis [65, 85]. Based on this knowledge, we conclude that the ossification of the lacrimal bone in *Qinglongtriton* was terminated before complete ossification of the lacrimal canal; this documents a previously unknown pattern of the canal in neotenic salamanders. All living salamanders that are obligate neotenes (cryptobranchids, proteids, amphiumids, sirenids) lack both the lacrimal bone and the nasolacrimal duct [16, 55, 72]. This seems to fit with the pattern in other amphibians in which aquatic forms tend to lose the eye gland and nasolacrimal duct [86, 87]. However, the discovery that neotenic *Beiyanerpeton* possesses a lacrimal [8] is in keeping with the demonstration that *Qinglongtriton* is another neotenic form with clear evidence of a nasolacrimal duct; hence, the apparent loss of a lacrimal bone and nasolacrimal duct in extant obligate neotenes is a specialization that is not a necessary consequence of failure to metamorphose.

In those salamanders having a nasolacrimal duct but lacking a lacrimal bone (ambystomatids, salamandrids, plethodontids), the prefrontal and maxilla take over the roles of the lacrimal to carry the nasolacrimal duct. These include both neotenic (*Ambystoma mexicanum*) and metamorphosed (salamandrids and plethodontids) forms. Medvedeva [88, 89] hypothesized that the prefrontal in these salamanders is actually a compound bone, the ‘prefronto-lacrimal’. Such a hypothesis seems to be supported by the *Ambystoma mexicanum*, in which the prefrontal may be ossified from two anlages [65]. A more recent study by Vater [90] showed that the prefrontal in the salamandrid *Triturus* is indeed a bone of dual origin, specifically the lacrimal and prefrontal.

#### Palatine as a Discrete Element

The presence of a toothed palatine as a discrete element in the adult stage is an unusual feature in salamanders. With the exception of sirenids, in most other salamanders the palatine occurs only in early ontogeny, but is subsequently lost by resorption [65] or fusion with the pterygoid [55, 91] at metamorphosis. Exceptions are also known from *Cryptobranchus* and *Amphiuma*, which show no trace of a palatine during the embryonic and larval stages [55]. Besides the recent report of *Beiyanerpeton* [8], *Qinglongtriton* is another Late Jurassic salamander that retained a palatine bone into the adult stage (Figs 3 and 6). Because the small palatine in these two Jurassic salamanders is free, rather than part of the palatopterygoid, this element could be resorbed, but not be fused to the pterygoid if these salamanders had proceeded metamorphosis. It appears that retention of a discrete palatine in these early salamanders likely resulted from delayed, incomplete resorption of the element, an ontogenetic pattern previously unknown for fossil or extant salamanders.

Embryonically in salamanders, the common developmental pattern is ossification of the palatine as a small plate connected to the anterior part of the palatopterygoid. At metamorphosis, the palatine along with anterior part of the pterygoid is resorbed [55, 65, 92]. This pattern is known for hynobiids, salamandrids, ambystomatids, plethodontids, and proteids [55, 65]. However, sirenids display a different pattern as has been documented in *Siren intermedia* by Reilly and Altig [93]. According to these authors, early occurrence of the palatine in *Siren* at stage I was not much different from other salamanders, but at stage II the palatine was freed from the pterygoid as a consequence of resorption of the latter element (palatopterygoid disintegration). Then, the resorption of the pterygoid continued to stage III, in which the pterygoid was completely lost, whereas the palatine persisted throughout ontogeny [93].

Because no metamorphosed salamanders are known with a palatine bone in adult stage, retention of the palatine in the two Jurassic salamandroids may be related to their neotenic condition; however, this does not mean that the feature characterizes neoteny, as many other neotenic salamanders lack a palatine as adults. Furthermore, the palatine cannot be detected even in early embryonic stages in cryptobranchids as neotenic salamanders [55]. Retention of the palatine in *Beiyanerpeton* and *Qinglongtriton* obviously reflects a distinct developmental feature different from all other known salamanders.

#### Stapes and Stapedial Foramen

Among extant salamanders, a stapes is present in all family groups except salamandrids [55], neotenic species of plethodontids [59], and the sirenid *Pseudobranchus* [55]. The lack of a stapes in all salamandrids is obviously a synapomorphy of the family, whereas that the similar condition in other two groups is probably independently acquired based on our knowledge of the phylogeny of Urodela [8].

The stapes in *Qinglongtriton* is penetrated by a stapedial foramen (Fig 6A; S1 Fig; S3 Movie), different from all living salamanders with a stapes, in which the foramen is entirely lost [55, 64, 72]. Our re-examination of specimens of *Beiyanerpeton jianpingensis* (e.g., PKUP V0601–V0603) indicates that the foramen is also present in that taxon. Absence of a stapedial foramen was regarded as a synapomorphy of the Batrachia [94]. On the contrary, presence of this foramen in Jurassic crown-group salamanders refutes this notion. Because the stem caudate *Karaurus* also has the stapes perforated [61, 95], the lack of a stapedial foramen in extant salamanders is re-interpreted here as a derived feature acquired within the crown-group salamanders. This is yet another example in which paleontological evidence reveals deep-time evolution of a particular feature that cannot be detected based on extant taxa only.

#### Fusion of Angular to Prearticular

Salamanders in Hynobiidae and Cryptobranchidae have a separate angular in the lower jaw. Loss of the angular by fusion with the prearticular is a derived feature that is diagnostic of the salamandroid clade. Multiple specimens of *Qinglongtriton* show a varied extent of fusion of the angular to the prearticular as has been described above. Because some of the relatively large specimens including the holotype (PKUP V0226, V0237, V0254, V0257) have the angular partly or fully fused to the prearticular along a visible sutural line (Fig 7C–7F), we conclude that the ontogenetic fusion of the angular to the prearticular in *Qinglongtriton* was delayed to a late developmental stage, even close to maturity.

In all known extant or extinct salamanders, a separate angular is only present in cryptobranchoids and their closely related fossil forms such as *Chunerpeton* [96]. Gardner [97] interpreted the presence of an angular in a Paleocene amphiumid (genus and species indeterminate) based on a notch on the posterolingual edge of the subdental shelf in an incomplete dentary (UALVP 14316). However, this interpretation cannot be warranted, because the angular, if present, does not articulate with the subdental shelf of the dentary, but more ventrally between the prearticular and the ventral edge of dentary. Instead, the notch that Gardner [97] described was likely for articulation with the prearticular.

#### Teeth and Crown Patterns

Pedicellate teeth with bicuspid crowns are two features that are usually used to characterize the Lissamphibia following the work of Parsons and Williams [98]. Although many caecilians [99, 100], and some salamanders and frogs have monocuspid and even non-pedicellate teeth, many developmental studies have documented the ontogenetic transition of teeth from a monocuspid and non-pedicellate larval dentition to a bicuspid and pedicellate adult dentition as a general pattern [98, 101–104]. For example, a study of tooth development in *Amphiuma* showed that the non-pedicellate and monocuspid larval teeth were replaced by pedicellate and bicuspid teeth as adult dentition [105]. Another study implied that the monocuspid crown pattern in some caecilians, larval salamanders, and some adult frogs is probably an ancestral condition, whereas the bicuspid condition is derived [106].

In extant salamanders, *Siren* has non-pedicellate teeth with monocuspid tooth-crowns, whereas the closely related *Pseudobranchus* has pedicellate teeth and monocuspid crowns [71, 98, 102, 107–109]. The teeth of proteids have been described as monocuspid and pedicellate in *Necturus* [108, 109], and those in *Proteus* as having no crown/base constriction [110] or having slight indication of a crown/base constriction [111]. Carroll and Holmes [63] concluded that: “the generally poor development of pedicelly in sirenids and proteids may be attributed to their neotenic nature.”

No bicuspid teeth have been found in specimens of *Qinglongtriton*. However, both marginal and palatal teeth are pedicellate, with a dividing zone separating the crown from the pedicel (Fig 8). This pattern demonstrates that bicuspid crown as a derived feature was not developed in association with pedicelly in this new salamander. The monocuspid crown is shared with its sister taxon *Beiyanerpeton*, but pedicelly is not. Of stem caudates, tooth morphology is unknown for Middle–Late Jurassic *Karaurus* and *Urupia*, but Skutschas and Martin [62] were able to show that the Middle Jurassic *Kokartus* had non-pedicellate teeth with monocuspid crowns. Gao and Shubin [8] determined that the dentition in the basal salamandroid *Beiyanerpeton* is non-pedicellate and monocuspid; they also argued that pedicelly and bicuspid crowns are derived features within Urodela, a notion supported by our study of the new salamander in this paper.

#### Basibranchial II

**Fig 11:**
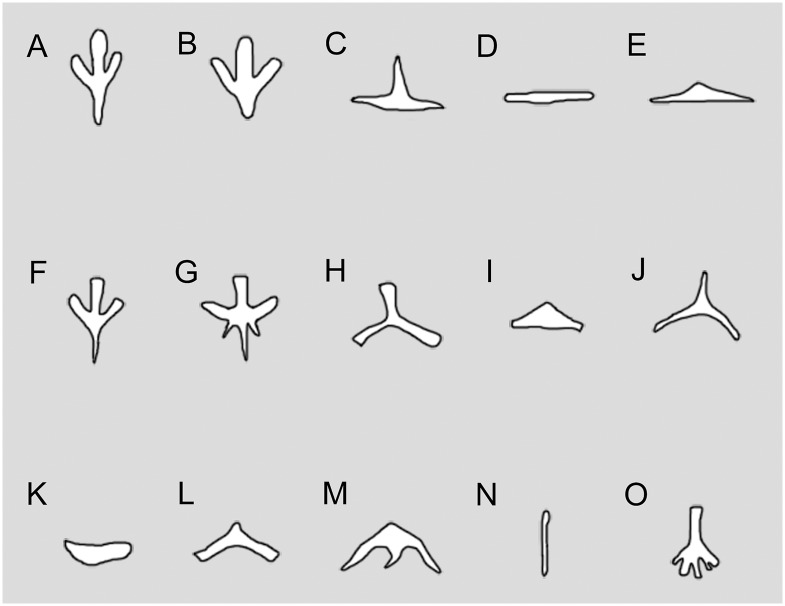
Comparison of basibranchial II of salamanders. A, *Karaurus* (Karauridae) based on Estes [61]; B, *Chunerpeton* (Cryptobranchidae) based on Gao and Shubin [96]; C, *Ranodon* (Hynobiidae); D, *Hynobius* (Hynobiidae); E, *Salamandrella* (Hynobiidae); C–E based on Xiong et al. [112]; F, G, juvenile and adult stage in *Qinglongtriton* (based on PKUP V0267 and PKUP V0226, respectively); H, *Valdotriton* (Salamandroidea) based on Evans and Milner [21]; I, *Salamandra* (Salamandridae); J, *Chioglossa* (Salamandridae); K, *Rhyacotriton* (Rhyacotritonidae); L, *Dicamptodon* (Dicamptodontidae); M, *Gyrinophilus* (Plethodontidae); I–M based on Wake and Deban [113]; N, *Proteus* (Proteidae) based on Rose [55]; O, *Siren* (Sirenidae) based on AmphibiaTree [114]. Not in scale.

Basibranchial II is the most posteromedian element in the hyobranchial apparatus of salamanders. The shape of the basibranchial II is highly variable among taxonomic groups [74]. In the stem caudate *Karaurus* and two Jurassic crown-group salamanders (*Chunerpeton* and *Beiyanerpeton*), basibranchial II is simply anchor-shaped, having a short median rod fused to a pair of anterolateral processes ([61, 96]; Fig 11A and 11B). The Early Cretaceous salamandroid *Valdotriton* from Spain has an inverted “Y”-shaped basibranchial II (Fig 11H). This configuration of the basibranchial II is basically similar to that in the European salamandrid *Chioglossa* (Fig 11J), and is likely a derived, rather than a primitive condition within the Salamandroidea (contra Evans and Milner [21]).

Among extant salamanders, a basibranchial II is absent in cryptobranchids [55], but is variably present or absent in hynobiids. Most hynobiids (*Ranodon*, *Pachyhynobius*, *Liua*, *Salamandrella*, and *Batrachuperus*) have a tri-radiate basibranchial II, with a horizontal bar fused to a short median process, so that the element is basically an inverted “T-shape” [112]. Two other hynobiids, *Hynobius* and *Pseudohynobius*, simply have a horizontal bar, lacking a median process, whereas basibranchial II is entirely absent in *Onychodactylus* [55, 82, 112].

Basibranchial II seems to be more variable in shape in salamandroids than in hynobiids. Most salamandrids (e.g., *Salamandrina*, *Pleurodeles*, *Triturus*, *Neurergus*, *Notophthalmus*, *Taricha*, *Cynops*, *Hypselotriton*, *Tylototriton*, *Pachytriton*, *Paramesotriton*) lack a basibranchial II at adult stage [82, 115], whereas those have it, it is either ossified as triangular plate as seen in *Salamandra* (Fig 11I) or an inverted Y-shaped structure as seen in *Chioglossa* (Fig 11J) and *Mertensiella* [116]. Dicamptodontids and ambystomatids have the tri-radiate condition, more or less resembling that in most hynobiids, whereas rhyacotritonids have a simple horizontal bar similar to other hynobiids. Amphiumids have basibranchial II entirely missing, whereas in proteids, it is a simple vertical bar (Fig 11N). In plethodontids, basibranchial II is basically anchor-shaped, but has all three rami oriented posteriorly as seen in *Gyrinophilus* (Fig 11M) and in *Eurycea* [59], or is a horizontal bar strongly bowed anteriorly without a posteromedial ramus as in *Hydromantes* [117]. Sirenids have a relatively long vertical stem, but is fused with a variable number of processes at the posterior end (Fig 11O). Overall, the presence or absence, or the shape of the basibranchial II are highly variable among salamanders, and no phylogenetic pattern can be unambiguously distinguished for this structure among salamander families.

Basibranchial II in mature individuals of *Qinglongtriton* (Fig 11G) differs from all known stem caudates and crown-group salamanders in having a median rod fused with a pair of anterolateral and a pair of posterolateral rami. Comparison of adult with juvenile specimens reveals ontogenetic change in hypobranchial II: small and presumably juvenile specimens (PKUP V0232, V0257, V0267; Fig 11F) display the primitive anchor-shaped basibranchial II, whereas large and presumably adult specimens (e.g., PKUP V0226, V0246, V0249, V0252) have added a second pair of processes posteriorly (Fig 11G). Moreover, ossification of the second pair of processes seems to be more extensive in keeping with increased body size.

Basibranchial II is the main force-transmitting element from the protractible musculature to the tongue pad [113]. It has been documented that basibranchial II provides the attachment for three muscles: M. geniohyoideus, M. rectus cervicis, and M. subarcualis oblique [118–121]. In those salamanders lacking basibranchial II (e.g., *Onychodactylus japonicus* and cryptobranchids), M. geniohyoideus is continuous with M. rectus cervicis, whereas M. subarcualis oblique inserts on the adjacent fascia of M. rectus cervicis [118, 119, 121]. In *Qinglongtriton*, the complex structure of basibranchial II may have strengthened the attachment of these muscles, thus enhancing the ability to control the hyobranchial apparatus during feeding and respiration.

#### Atlas and Anterior Trunk Vertebrae

Two notable features in the vertebral column of *Qinglongtriton* are worth discussion: a pair of transverse processes on the neural arch of the atlas and a median depression in the ventral surface of the centrum in the atlas and one or two anterior trunk vertebrae. The transverse processes on the atlas of *Qinglongtriton* are rudimentary, but are comparable to those in extant salamanders that have the processes. In crown-group salamanders, an atlas with rudimentary transverse process can be found in proteids, sirenids, and *Ambystoma gracile*, whereas the process is lacking in *Cryptobranchus* and *Amphiuma* [77, 97, 114]. To our knowledge, no metamorphosed salamanders are known with such processes. In stem caudates, the transverse process on the atlas can be found in *Kokartus* and *Urupia*, both have been recognized as neotenic [62, 76]. None of the taxa mentioned above have ribs associated with the atlas.

Rogers [122] claimed that the transverse process of the atlas is present in larval individuals of *Ambystoma tigrinum* but the process is reabsorbed at metamorphosis. Later workers argued that possession of transverse processes on the atlas is probably a neotenic feature [62, 123]. Our new fossil discovery, corroborated with evidence from other fossil and extant salamanders, appears to support the interpretation that presence of transverse processes on the atlas is indeed of a feature associated with neoteny with the exception of *Cryptobranchus* and *Amphiuma*.

The atlas of *Qinglongtriton* resembles to that of *Kokartus* and *Urupia* in having a median depression on the ventral surface (PKUP V0226, V0229, V0235, V0249, V0256, V0259). In addition, the first two trunk vertebrae in *Qinglongtriton* also bear a deep median depression on the ventral surface of the centrum without known functions. A median depression on the ventral surface of the atlas or anterior trunk vertebrae is also known in several extant taxa (*Siren*, *Andrias*, *Cryptobranchus*, *Necturus*, *Ambystoma*, *Amphiuma*, and *Eurycea*), and in a hynobiid species *Batrachuperus longdongensis* (CIB 20020489, 20020499). Because all these are neotenic salamanders, and no metamorphosed species have such a depression to our knowledge, it seems reasonable to interpret the presence of such a distinct depression in the atlas and the first two trunk vertebrae as another neotenic feature in addition to the rudimentary transverse process of the atlas as discussed above.

### Phylogenetic Placement of *Qinglongtriton*

**Fig 12:**
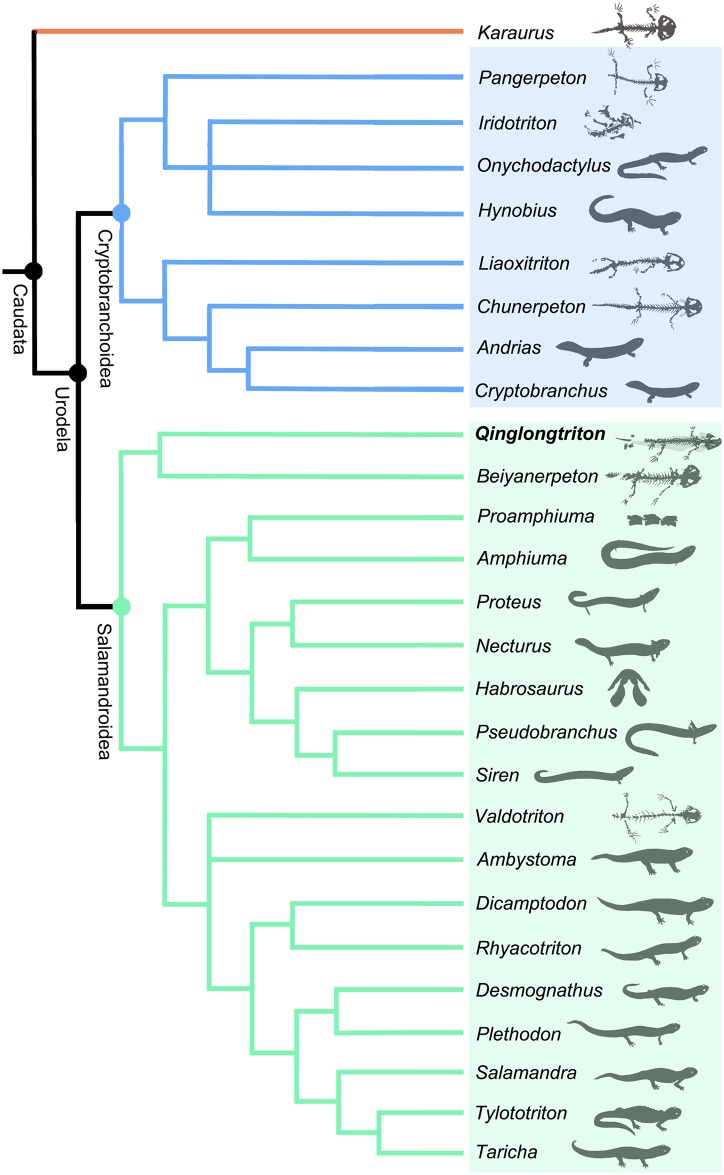
Strict consensus tree of three MPTs resulted from analysis of morphological dataset (S1 Appendix) using TNT (Goloboff et al. [124, 125]).

The data matrix used in this study is revised from Gao and Shubin ([8]: supplementary information appendix), with the new salamander *Qinglongtriton* added as the additional taxon. Revised character codings of some fossil taxa are made based on our examination of specimens at hand and are highlighted in the dataset (S1 Appendix). Of the 105 characters in the original dataset, 74 were scored for the new taxon (see S1 Appendix). As in the original analyses, the stem-caudate *Karaurus* was designated as the outgroup, and all characters were unordered and equally weighted. As in the original analysis [8], several fossil taxa (e.g., *Laccotriton*, *Jeholotriton*) were excluded from the analysis due to their anatomical uncertainties are under revision. Several characters (11, 18, 47, 50, 72, 73, 92, 93, 103) were excluded from the current analyses because these are uninformative in relation to the taxon sampling in the current dataset. The analysis of the revised dataset was performed using the implicit enumeration search option in TNT (Version 1.1; Goloboff et al. [124, 125]). Three most parsimonious trees (MPTs) were found (tree length = 241 steps, consistent index = 0.494, retention index = 0.725). As seen in the strict consensus tree (Fig 12), all the three MPTs have the new taxon placed as the sister taxon with *Beiyanerpeton*, and the two taxa together form the basalmost clade within the Salamandroidea. Thus, *Qinglongtriton* adds another salamandroid that documented the early evolution of the clade during the Late Jurassic in North China.

*Qinglongtriton* shares with *Beiyanerpeton* and other salamandroids derived character states as follows: nasals separated by anterodorsal fenestra without a midline contact (8–1); anterolateral process of parietal extending beyond midlevel of orbit; angular fused to prearticular (26–1); articular absent by fusion to prearticular (29–1); and spinal nerve foramina occur in trunk, sacral and caudal vertebrae (51–3). The sister-group relationship of *Qinglongtriton* with *Beiyanerpeton* within the Salamandroidea is supported by three characters: palatine in adult present as discrete element (19–0); pterygoid teeth present (39–1); and monocuspid tooth crown (42–0).

In general, the new taxon *Qinglongtriton* adds another rare fossil salamander to the Yanliao Biota in addition to the previous report of *Beiyanerpeton* from the same geological formation. The new discovery provides further support for the hypothesis that the split of the Salamandroidea from Cryptobranchoidea was deeply rooted in salamander phylogeny before the Late Jurassic (Oxfordian) time.

## Conclusions

The following conclusions can be drawn from our study of *Qinglongtriton gangouensis* from the Nanshimenzi locality:

1. *Qinglongtriton gangouensis* is a neotenic salamandroid named and described based on multiple specimens from the Upper Jurassic Tiaojishan Formation.
2. The new salamandroid displays several features of ontogenetic or taxonomic significance, including: lacrimal with a dorsally open groove for the nasolacrimal duct; presence of a stapedial foramen; lack of ossification of the orbitosphenoid; pedicellate teeth with monocuspid crowns; presence of a toothed coronoid; ontogenetic fusion of the angular bone to the prearticular; and a unique shape of the basibranchial II.
3. Phylogenetic analysis places *Qinglongtriton* as the sister taxon to *Beiyanerpeton*, and the two taxa together form the basalmost clade within the Salamandroidea. Along with the coexisting *Beiyanerpeton* the new discovery indicates that morphological disparity of the salamandroid clade was already underway by early Late Jurassic (Oxfordian) time.

## Supporting Information

S1 Appendix Taxon-character data matrix used in phylogenetic analysis (A = 0/1; B = 0/2; C = 1/2). Character description see Gao and Shubin (2012; 10.1073/pnas.1009828109/-/DCSupplemental). Modified coding of characters (bold-faced and underlined) as explained in text below.
(PDF)

S1 Fig CT-rendered reconstruction of skull of PKUP V0226 (holotype of *Qinglongtriton gangouensis* gen. et sp. nov.) in dorsal (left) and ventral (right) views. Abbreviations used as in Meta Data section.
(TIF)

S2 Fig Referred specimen of *Qinglongtriton gangouensis* gen. et sp. nov. (PKUP V0227). Photograph (left) and line drawing (right) of incomplete skeleton in dorsal view. Abbreviations used as in Meta Data section.
(TIF)

S3 Fig Referred specimen of *Qinglongtriton gangouensis* gen. et sp. nov. (PKUP V0254). Photograph (left) and line drawing (right) of incomplete skeleton in dorsal view. Abbreviations used as in Meta Data section.
(TIF)

S4 Fig Close-up photographs of referred specimens of *Qinglongtriton gangouensis* gen. et sp. nov., showing horizontal groove (arrows) and sensory foramina (sfm) in premaxilla. A, PKUP V0237; B, PKUP V0254.
(TIF)

S5 Fig Close-up photographs of referred specimens of *Qinglongtriton gangouensis* showing horizontal groove (arrows) and foramina laterale nasi (fln) in left maxilla. A, PKUP V0234; B, PKUP V0254.
(TIF)

S6 Fig Close-up photographs of referred specimens of *Qinglongtriton gangouensis* gen. et sp. nov., showing nasolacrimal groove (arrows) in lacrimal and prefrontal. A, PKUP V0237; B, PKUP V0254.
(TIF)

S7 Fig Close-up photographs of limbs in referred specimens of *Qinglongtriton gangouensis* gen. et sp. nov. A, PKUP V0245; B, PKUP V0251. Abbreviations used as in Meta Data section.
(TIF)

S1 Movie Reconstruction of the upper body of PKUP V0226 (holotype of *Qinglongtriton gangouensis* gen. et sp. nov.). (MP4)

S2 Movie Reconstruction of whole body of PKUP V0228 (referred specimen of *Qinglongtriton gangouensis* gen. et sp. nov.). (MP4)

S3 Movie CT rendered reconstruction of skull of PKUP V0226 (holotype of *Qinglongtriton gangouensis* gen. et sp. nov.). (MP4)

## Abbreviations

CIB: Chengdu Institute of Biology, Chinese Academy of Sciences, Chengdu, China
PKUP: Peking University Paleontological Collections, Beijing, China
UALVP: University of Alberta, Laboratory for Vertebrate Paleontology, Edmonton, Canada
adf: anterodorsal fenestra
amf: anteromedial fenestra
anf: angular foramen
at: atlas
bb: basibranchial
co: coronoid
d: dentary
exo: exoccipital
fe: femur
fi: fibula
fr: frontal
gr: gill raker
hb: hypobranchial
hu: humerus
icaf: internal carotid foramen
il: ilium
isc: ischium
lac: lacrimal
m: maxilla
na: nasal
opi: opisthotic
pa: parietal
pal: palatine
pm: premaxilla
pra: prearticular
praf: prearticular foramen
prf: prefrontal
pro: prootic
ps: parasphenoid
pt: pterygoid
qu: quadrate
ra: radius
sca: scapulocoracoid
sm: septomaxilla
sq: squamosal
st: stapes
ti: tibia
ul: ulna
vo: vomer
