# Supplementary material for: A New Basal Salamandroid (Amphibia, Urodela) from the Late Jurassic of Qinglong, Hebei Province, China
Source: PLoS One. 2016 May 4;11(5):e0153834. doi: 10.1371/journal.pone.0153834 (PMC4856324; doi:10.1371/journal.pone.0153834)
Supplement: S1 Appendix — Character description see Gao and Shubin (2012; 10.1073/pnas.1009828109/-/DCSupplemental). Modified coding of characters (bold-faced and underlined) as explained in text below. (PDF) [file pone.0153834.s001.pdf]

A New Basal Salamandroid (Amphibia, Urodela) from the Late Jurassic of Qinglong, Hebei Province, China

Supporting Information

Jia Jia, Ke-Qin Gao

Correspondence author: Ke-Qin Gao (email:kqgao@pku.edu.cn)

S1 Appendix. Taxon-character data matrix used in phylogenetic analysis (A= 0/1; B= 0/2; C= 1/2). Character description see Gao and Shubin (2012; doi:10.1073/pnas.1009828109/-/DCSupplemental). Modified coding of characters (bold-faced and underlined) as explained in text below.

					5					10					15					20					25					30
Karaurus	0	0	0	?	?	0	0	0	0	0	0	?	0	0	0	0	0	0	?	0	0	0	0	0	?	0	?	?	0	0
Cryptobranchus	0	0	0	0	1	1	1	0	1	1	1	1	1	1	0	1	0	0	1	0	0	0	1	0	0	0	1	?	1	0
Andrias	0	0	0	0	1	1	1	0	1	1	1	1	1	1	0	1	0	0	1	0	0	0	1	0	0	0	1	?	1	0
Hynobius	0	0	0	A	0	A	0	0	0	0	1	1	A	1	0	0	0	0	1	0	A	0	0	A	0	0	1	?	0	0
Onychodactylus	0	0	0	0	0	0	0	0	0	0	1	1	1	1	0	0	0	0	1	0	0	0	0	A	0	0	1	?	0	0
Plethodon	A	1	0	0	0	0	0	1	0	1	1	3	1	2	1	?	?	?	1	0	1	0	0	0	1	1	1	?	1	0
Desmognathus	1	1	0	0	0	0	0	1	0	1	1	3	1	2	1	?	?	?	1	0	1	1	?	?	1	1	1	?	1	0
Amphiuma	1	2	0	0	1	1	1	1	0	1	1	3	1	0	0	2	0	0	1	0	1	0	0	0	0	1	1	?	1	0

Proamphiuma	?	?	?	?	?	?	?	?	?	?	?	?	?	?	?	?	?	?	?	?	?	?	?	?	?	?	?	?	?	?
Rhyacotriton	0	1	0	0	0	0	0	1	0	0	1	1	1	2	0	0	0	0	1	0	0	0	0	1	0	1	1	?	0	0
Siren	0	3	1	?	1	0	1	1	1	1	1	2	0	0	0	2	0	0	0	1	1	1	?	?	1	1	0	**1**	A	1
Pseudobranchus	0	3	1	?	1	0	1	1	1	1	1	2	0	0	0	2	0	0	0	1	1	1	?	?	1	1	0	**1**	1	1
Habrosaurus	0	?	0	?	?	?	?	?	?	?	?	?	0	0	?	?	?	?	0	?	?	?	?	?	?	?	?	?	?	0
Necturus	0	1	1	?	1	1	0	2	?	1	1	0	1	0	0	2	1	0	1	0	1	1	?	?	1	1	0	1	1	0
Proteus	0	1	1	?	1	1	0	2	?	1	1	0	1	0	0	2	1	0	1	0	1	1	?	?	1	1	0	1	1	0
Salamandra	0	1	0	1	1	0	0	1	0	1	1	3	2	2	0	0	1	0	1	0	1	0	0	0	0	1	1	?	0	0
Taricha	1	1	0	1	1	0	0	1	0	1	1	3	2	1	0	0	1	0	1	0	1	0	0	0	0	1	1	?	0	0
Tylototriton	0	0	0	1	1	0	0	0	0	1	1	3	2	1	0	0	1	A	1	0	1	0	0	0	0	1	1	?	0	0
Dicamptodon	0	1	0	0	0	0	0	1	0	0	1	3	A	B	0	0	A	0	1	0	0	0	0	0	0	1	0	0	1	0
Ambystoma	0	1	0	0	0	1	0	1	0	1	1	3	0	2	0	0	0	0	1	0	A	0	0	0	0	1	1	?	A	0
Valdotriton	0	0	0	0	?	1	1	0	0	1	1	?	0	0	0	0	?	?	1	0	1	0	0	?	?	1	1	?	1	0
Jeholotriton	0	0	0	?	?	0	0	0	0	?	1	?	1	?	0	0	0	0	?	0	?	?	?	?	?	0	1	?	?	0
Liaoxitriton	0	0	0	0	?	0	0	0	0	?	1	0	1	1	0	0	0	0	1	0	1	0	0	0	?	0	1	?	0	0
Pangerpeton	0	?	0	?	?	?	0	?	?	?	1	0	2	?	0	0	0	0	?	0	0	?	?	?	?	1	1	?	?	0
Chunerpeton	0	0	0	0	?	0	0	0	1	1	1	0	0	0	0	0	0	0	?	0	0	0	1	0	?	0	1	?	0	0
Beiyanerpeton	0	0	0	0	0	0	0	1	0	0	1	0	0	0	0	0	0	0	0	0	1	0	0	1	?	1	1	?	1	0
Iridotriton	0	0	0	?	?	?	0	?	?	?	1	?	?	?	?	?	?	?	?	?	?	0	?	?	?	0	1	?	?	0
Qinglongtriton	0	0	0	0	0	0	0	1	0	0	1	0	0	1	0	0	0	0	0	0	0	0	0	0	?	1	0	1	1	0

					35					40					45					50					55					60
Karaurus	0	0	0	0	0	0	0	**?**	?	0	?	?	?	0	0	0	1	0	0	0	?	0	?	?	?	?	?	0	0	0
Cryptobranchus	0	A	1	0	1	0	0	**?**	0	0	**1**	1	0	1	0	0	1	1	0	1	0	0	1	0	0	0	1	1	1	1
Andrias	0	1	1	0	1	0	0	**?**	0	0	**1**	1	0	1	0	0	1	1	0	1	0	0	1	0	0	0	1	1	1	1
Hynobius	0	1	1	0	1	0	C	**?**	0	0	**1**	1	0	0	0	0	1	1	0	1	0	0	0	0	0	0	0	1	0	**A**
Onychodactylus	0	A	1	0	1	0	1	**?**	0	0	**1**	1	0	0	0	0	1	1	0	1	0	0	1	0	0	0	0	1	0	0
Plethodon	0	0	1	1	0	0	1	**?**	?	1	**1**	1	0	?	0	1	1	0	0	1	3	0	1	1	1	0	0	1	0	0
Desmognathus	0	0	1	1	0	0	1	**?**	?	1	**1**	1	0	?	0	1	1	0	0	1	3	0	1	1	1	0	0	1	0	0
Amphiuma	0	1	0	0	1	0	0	**?**	0	0	**1**	1	0	1	0	0	1	0	1	1	1	0	1	1	0	1	1	A	0	1
Proamphiuma	?	?	?	?	?	?	?	?	?	?	?	?	?	?	0	0	1	0	?	1	1	?	?	?	?	?	?	?	?	?
Rhyacotriton	0	0	1	1	0	0	1	**?**	0	0	**1**	1	0	2	0	0	1	0	0	1	2	0	1	1	1	0	0	1	0	1
Siren	1	0	0	0	0	1	3	1	0	0	**0**	0	0	2	1	0	1	0	1	1	3	0	1	1	0	1	1	1	1	1
Pseudobranchus	1	0	0	0	0	1	3	1	0	0	**1**	0	0	1	1	0	1	0	1	1	3	1	1	1	0	1	1	1	1	1
Habrosaurus	1	?	?	?	?	0	0	1	?	?	**0**	0	?	?	1	0	1	?	?	**1**	**3**	?	?	?	?	?	?	?	?	?
Necturus	0	0	1	0	0	0	1	**?**	1	0	**1**	0	1	1	0	0	1	0	0	1	0	0	1	1	0	1	1	1	?	1
Proteus	0	0	0	0	0	0	?	**?**	1	0	**0**	0	1	1	0	0	1	0	0	1	0	0	1	1	0	1	1	1	?	1
Salamandra	0	1	1	1	0	0	2	**?**	0	0	**1**	1	0	2	0	1	1	0	0	1	3	1	0	1	1	0	0	1	0	0
Taricha	0	0	0	1	1	0	2	**?**	0	0	**1**	1	0	2	0	1	1	0	0	1	3	1	0	1	1	0	0	1	0	0
Tylototriton	0	0	0	1	1	0	2	**?**	0	0	**1**	1	0	2	0	1	1	0	0	1	3	1	0	1	1	0	0	0	0	0
Dicamptodon	0	0	A	1	0	0	A	**?**	A	0	**1**	1	0	0	0	0	1	0	0	1	2	0	0	1	1	0	0	1	0	1
Ambystoma	0	0	1	1	0	0	0	**?**	0	0	**1**	1	0	2	0	0	1	0	0	1	3	0	0	1	1	0	0	1	0	1

Valdotriton	0	?	1	1	0	0	0	**?**	0	0	**1**	?	0	2	0	0	1	0	0	1	2	0	?	?	?	?	?	1	0	1
Jeholotriton	0	?	?	?	?	0	?	?	?	0	?	?	?	2	0	0	?	1	0	?	?	0	?	?	?	?	?	1	0	0
Liaoxitriton	0	0	0	0	?	0	0	**?**	0	0	**1**	?	**0**	0	0	0	?	1	0	?	?	0	?	?	?	?	?	1	0	0
Pangerpeton	0	**0**	**0**	**?**	**0**	0	1	**?**	0	0	?	?	0	?	0	0	1	1	0	1	?	?	?	?	?	?	?	**1**	?	?
Chunerpeton	0	0	0	?	0	0	0	**?**	0	0	?	?	0	?	0	0	?	1	0	?	?	0	?	?	?	?	?	1	1	1
Beiyanerpeton	0	?	0	?	0	0	0	0	1	0	**0**	0	0	0	0	0	?	0	0	1	?	0	0	?	?	?	?	1	0	1
Iridotriton	?	?	?	?	?	0	?	?	?	?	1	?	?	?	0	0	?	1	0	?	?	?	?	?	?	?	?	1	?	0
Qinglongtriton	0	0	0	?	0	0	0	0	1	0	1	0	1	0	0	0	1	0	0	1	3	0	1	?	?	?	0	1	0	1

					65					70					75					80					85					90
Karaurus	0	0	0	0	?	0	0	?	0	0	0	?	?	?	?	?	?	?	?	?	?	?	?	?	?	?	?	?	?	?
Cryptobranchus	1	1	0	1	0	0	2	0	0	0	0	1	1	0	0	A	0	0	0	0	0	0	2	0	0	0	0	0	1	1
Andrias	1	1	0	1	0	0	2	0	0	0	0	1	1	A	0	1	0	0	0	0	0	0	2	0	0	0	0	0	1	1
Hynobius	0	0	0	1	A	A	2	0	1	1	0	1	1	A	1	0	0	0	0	0	?	0	2	0	0	0	0	0	1	1
Onychodactylus	0	0	0	1	1	1	2	0	1	1	0	1	1	1	0	1	0	0	0	0	?	0	2	0	0	0	0	0	1	1
Plethodon	0	0	A	2	1	1	2	1	1	?	?	1	1	1	0	1	2	2	1	1	1	0	2	1	2	1	1	1	0	1
Desmognathus	0	1	0	2	1	1	2	1	1	?	?	1	1	1	0	?	2	2	1	1	1	0	2	A	2	1	1	1	0	1
Amphiuma	1	0	1	2	1	0	2	1	0	0	0	1	1	2	A	1	2	2	1	0	A	0	1	1	1	0	1	0	0	1
Proamphiuma	?	?	?	?	?	?	?	?	?	?	?	?	?	?	?	?	?	?	?	?	?	?	?	?	?	?	?	?	?	?
Rhyacotriton	0	0	0	2	A	1	2	1	0	0	0	1	1	1	1	0	2	2	1	1	1	1	1	1	1	1	1	1	0	1
Siren	?	0	1	2	1	1	1	1	0	0	?	1	?	1	1	1	2	1	1	2	1	0	0	0	0	0	0	0	?	0

Pseudobranchus	?	0	1	1	1	1	1	1	0	0	?	1	?	1	1	1	2	1	1	2	1	0	0	0	0	0	0	0	?	0
Habrosaurus	?	?	?	?	?	?	?	?	?	?	?	?	?	?	?	?	?	?	?	?	?	?	?	?	?	?	?	?	?	?
Necturus	?	?	1	2	0	0	2	1	0	0	0	1	1	2	1	?	1	2	1	1	1	0	2	1	1	1	1	1	0	1
Proteus	?	?	1	1	0	0	2	1	0	0	0	1	1	2	1	?	1	2	1	1	1	0	2	1	1	1	1	1	0	1
Salamandra	0	0	0	2	1	1	2	0	1	1	1	1	1	1	1	1	2	2	1	0	0	0	2	1	1	1	1	0	0	1
Taricha	0	0	1	2	1	1	2	0	1	1	1	1	1	1	1	1	2	2	1	0	0	0	2	1	1	1	1	0	0	1
Tylototriton	0	0	1	1	1	0	2	0	1	1	1	1	1	0	1	1	2	2	1	0	0	0	2	1	1	0	1	0	0	1
Dicamptodon	**0**	0	0	2	1	1	2	0	1	1	0	1	1	1	0	0	2	2	1	0	0	1	2	1	1	1	1	1	0	1
Ambystoma	1	0	0	2	1	1	2	0	0	0	0	1	1	1	0	0	2	2	1	0	0	1	2	1	2	1	1	1	0	1
Valdotriton	?	0	0	2	1	1	?	?	1	?	0	?	?	?	?	?	?	?	?	?	?	?	?	?	?	?	?	?	?	?
Jeholotriton	0	0	0	1	1	0	?	?	0	?	0	?	?	?	?	?	?	?	?	?	?	?	?	?	?	?	?	?	?	?
Liaoxitriton	0	0	0	?	?	0	?	?	1	?	0	1	1	0	0	?	?	?	?	?	?	?	?	?	?	?	?	?	?	?
Pangerpeton	?	?	?	?	?	?	?	?	0	0	0	?	?	?	?	?	?	?	?	?	?	?	?	?	?	?	?	?	?	?
Chunerpeton	0	0	0	1	1	0	?	?	0	0	0	?	?	?	?	?	?	?	?	?	?	?	?	?	?	?	?	?	?	?
Beiyanerpeton	1	0	0	**0**	?	0	?	?	**0**	0	0	?	?	?	?	?	?	?	?	?	?	?	?	?	?	?	?	?	?	?
Iridotriton	?	?	?	?	?	?	?	?	1	1	?	?	?	?	?	?	?	?	?	?	?	?	?	?	?	?	?	?	?	?
Qinglongtriton	0	0	0	0	1	0	?	?	0	0	0	?	?	?	?	?	?	?	?	?	?	?	?	?	?	?	?	?	?	?

					95					100					105
Karaurus	0	1	1	1	?	?	?	?	0	?	?	0	0	?	?
Cryptobranchus	1	1	1	1	0	0	0	0	0	1	0	0	0	0	0

Andrias	1	1	1	1	0	0	0	0	0	1	0	0	0	0	?
Hynobius	1	1	1	1	1	0	0	0	0	0	0	0	0	0	?
Onychodactylus	1	1	1	1	1	0	0	2	0	?	0	0	0	0	?
Plethodon	2	1	1	1	1	1	1	2	0	1	0	0	0	?	0
Desmognathus	2	1	1	1	1	1	1	2	0	1	0	0	0	?	0
Amphiuma	2	1	1	1	0	0	1	0	1	1	0	0	0	?	0
Proamphiuma	2	?	?	?	?	?	?	?	?	?	?	?	?	?	?
Rhyacotriton	2	1	1	1	1	0	1	1	0	1	0	0	0	?	?
Siren	2	1	1	0	0	0	0	0	1	1	1	0	0	0	0
Pseudobranchus	2	1	1	0	0	0	0	0	1	1	1	0	0	?	?
Habrosaurus	?	?	?	?	?	?	?	?	?	?	?	?	?	?	?
Necturus	2	1	1	1	0	0	1	0	0	1	0	0	0	1	1
Proteus	2	1	1	1	0	0	1	0	1	1	0	0	0	1	1
Salamandra	2	1	1	1	1	0	1	0	0	1	0	0	0	?	0
Taricha	2	1	1	1	1	0	1	0	0	1	0	1	0	?	?
Tylototriton	2	1	1	1	1	0	1	0	0	1	0	1	1	?	?
Dicamptodon	2	1	1	1	1	0	1	0	0	1	0	0	0	0	?
Ambystoma	2	1	1	1	A	0	1	0	0	1	0	0	0	?	0
Valdotriton	2	1	1	1	?	?	?	?	0	?	?	0	0	?	?
Jeholotriton	?	1	1	1	?	?	?	?	0	?	?	0	0	?	?
Liaoxitriton	**1**	1	1	1	?	?	?	?	0	?	0	0	0	0	?
Pangerpeton	**1**	1	1	1	?	?	?	?	0	?	?	0	0	?	?

Chunerpeton	1	1	1	1	?	?	?	?	0	?	0	0	**0**	?	?
Beiyanerpeton	1	1	1	1	?	0	?	?	0	?	0	0	**0**	0	?
Iridotriton	?	?	?	1	?	?	?	?	0	?	0	?	?	?	?
Qinglongtriton	1	1	1	1	?	0	?	?	0	?	0	0	0	0	?

Modification of character coding:

Character 28. Coronoid teeth in adult: absent (0); present (1):

Siren and *Pseudobranchus* were incorrectly coded as (0) in Gao and Shubin (2012: SI table 1), but are re-coded as (1) based on information from Clemen and Greven (1988), Gardner (2003), and Rose (2003).

Character 32: Hypobranchial I and ceratobranchial I: two elements remain separate (0); two elements fused (1):

Pangerpeton was coded as “?” in Gao and Shubin (2012), but is re-coded as “0” based on our observation of specimens in PKUP collections.

Character 33. Ossification of hypobranchial I: present (0); absent (1).

This character was incorrectly coded (1) for *Pangerpeton* in Gao and Shubin (2012), but has been re-coded as (0) in agreement with remarks on character 32 in Gao and Shubin (2012).

Character 34. Ceratobranchial II in adults: present (0); absent (1).

Pangerpeton was coded as (0) in Gao and Shubin (2012), but has been re-coded as unknown (?) in agreement with remarks on this character in Gao and Shubin (2012).

Character 35: Basibranchial II: present as ossified or cartilaginous (0); or absent (1).

Pangerpeton was coded as “1” in Gao and Shubin (2012), but is re-coded as “0” based on our observation of specimens in PKUP collections.

Character 38. Multiple and parallel rows of palatine teeth in adult: absent (0); present (1):

In keeping with the statement made in Gao and Shubin (2012: Supplementary Information Pp. 13), all taxa that lack a discrete palatine in adults have been re-coded as inapplicable in the revised dataset.

Character 41. Marginal teeth: nonpedicellate (0); pedicellate (1):

All taxa but *Iridotriton* were coded with opposite character polarity against description in the original dataset (Gao and Shubin, 2012). We have reversed the coding of this character for those taxa involved in the revised dataset: (0) changed to (1), and (1) to (0). *Siren* is coded as (0) based on Clemen and Greven (1988) and Gardner (2003).

Character 43. Sphenethmoid as a discrete bone: present (0); absent (1).

Liaoxitriton was coded as (?), but has been re-coded as (0) based on our observation of specimens in PKUP collections.

Character 50. Atlantal spinal nerve foramen: absent (0); present (1):

Habrosaurus was coded as unknown (?) in original dataset (Gao and Shubin, 2012), but has been re-coded as (1) based on information from Gardner (2003).

Character 51. Postatlantal spinal nerve foramina: all postatlantal spinal nerves exit intervertebrally (0); spinal nerve foramina present on posterior caudal vertebrae (1); foramina present on all caudal vertebrae (2); foramina occur in trunk, sacral and caudal series (3):

Habrosaurus was coded as unknown (?), but has been re-coded as (3) based on information from Gardner (2003).

Character 58. Sculptured dermal skull roof: present as heavily sculptured surface covering (0); weakly sculptured or absent (1).

Pangerpeton was coded as unknown (?), but has been re-coded as (1) based on our observation of specimens in PKUP collections.

Character 60. Anterolateral process of parietal: poorly defined or absent (0); well-developed process extending to or surpassing midlevel of orbit (1):

Hynobius was coded (0) in Gao and Shubin (2012), but has been re-coded as (0/1) based on information from Sato (1943: *Hynobius tokyoensis* has a long process).

Character 61. Parietal/prefrontal contact above orbit: contact absent (0); contact present to embrace frontals (1):

Dicamptodon was coded as (1) in Gao and Shubin (2012), but has been re-coded as (0) based on Wake (2001).

Character 64. Exposure of otic-occipital complex in dorsolateral view: otic-occipital complex largely concealed by parietal (0); mainly exposed posterior to parietal (1); large exposure extends lateral to parietal bone (2).

Beiyanerpeton was coded as unknown (?) in Gao and Shubin (2012), but has been re-coded as (0) in keeping with information from the original description of the taxon (Gao and Shubin, 2012).

Character 69. Dorsal and ventral crests of humerus: poorly defined (0); well developed (1).

Beiyanerpeton was coded as unknown (?) in original dataset, but has been re-coded as (0) based on our observation of newly prepared specimens.

Character 91. Number of free ribs on anterior caudal vertebrae: more than three pairs (0); two to three pairs (1); or free ribs absent (2).

Liaoxitriton and *Pangerpeton* were coded as unknown (?) in Gao and Shubin (2012), but has been re-coded as (1) based on our observation of specimens in PKUP collections.

Character 103. Maxillary arcade: incomplete (0); complete (1).

Chunerpeton and *Beiyanerpeton* were inaccurately coded as unknown (?) in the published dataset by Gao and Shubin (2012), but are corrected here as (0) based on their lack of a jugal bone.

Literature Cited:

Clemen G, Greven H. Morphological studies on the mouth cavity of Urodela IX. Teeth of the palate and the splenials in *Siren* and *Pseudobranchus* (Sirenidae: Amphibia). Sonderdruck aus Zeitschrift für zoologische Systematik und Evolutionsforschung. 1988;26: 135–143.

Gao KQ, Shubin NH. Late Jurassic salamandroid from western Liaoning, China. Proceedings of the National Academy of Sciences of the United States of America. 2012;109: 5767–5772.

Gardner JD. Revision of *Habrosaurus* Gilmore (Caudata; Sirenidae) and relationships among sirenid salamanders. Palaeontology. 2003;46: 1089–1122.

Rose CS. The developmental morphology of salamander skulls. In: Heatwole H, Davies M, editors. Amphibian Biology. Volume 5: Osteology. Chipping Norton, New South Wales: Surrey Beatty and Sons; 2003. pp. 1684–1783.

Sato I. A monograph of the tailed batrachians of Japan. Osaka: Nippon Shuppan-sha; 1943.

Wake DB. "*Dicamptodon ensatus*", Digital Morphology. 2001. Available: http://digimorph.org/specimens/*Dicamptodon_ensatus*/. Accessed 29 April 2015.

Wang Y, Evans SE. A new short-bodied salamander from the Upper Jurassic/Lower Cretaceous of China. Acta Palaeontologica Polonica. 2006;51: 127–130.
