# Supplementary figures and images for: A New Basal Salamandroid (Amphibia, Urodela) from the Late Jurassic of Qinglong, Hebei Province, China
Source: PLoS One. 2016 May 4;11(5):e0153834. doi: 10.1371/journal.pone.0153834 (PMC4856324; doi:10.1371/journal.pone.0153834)

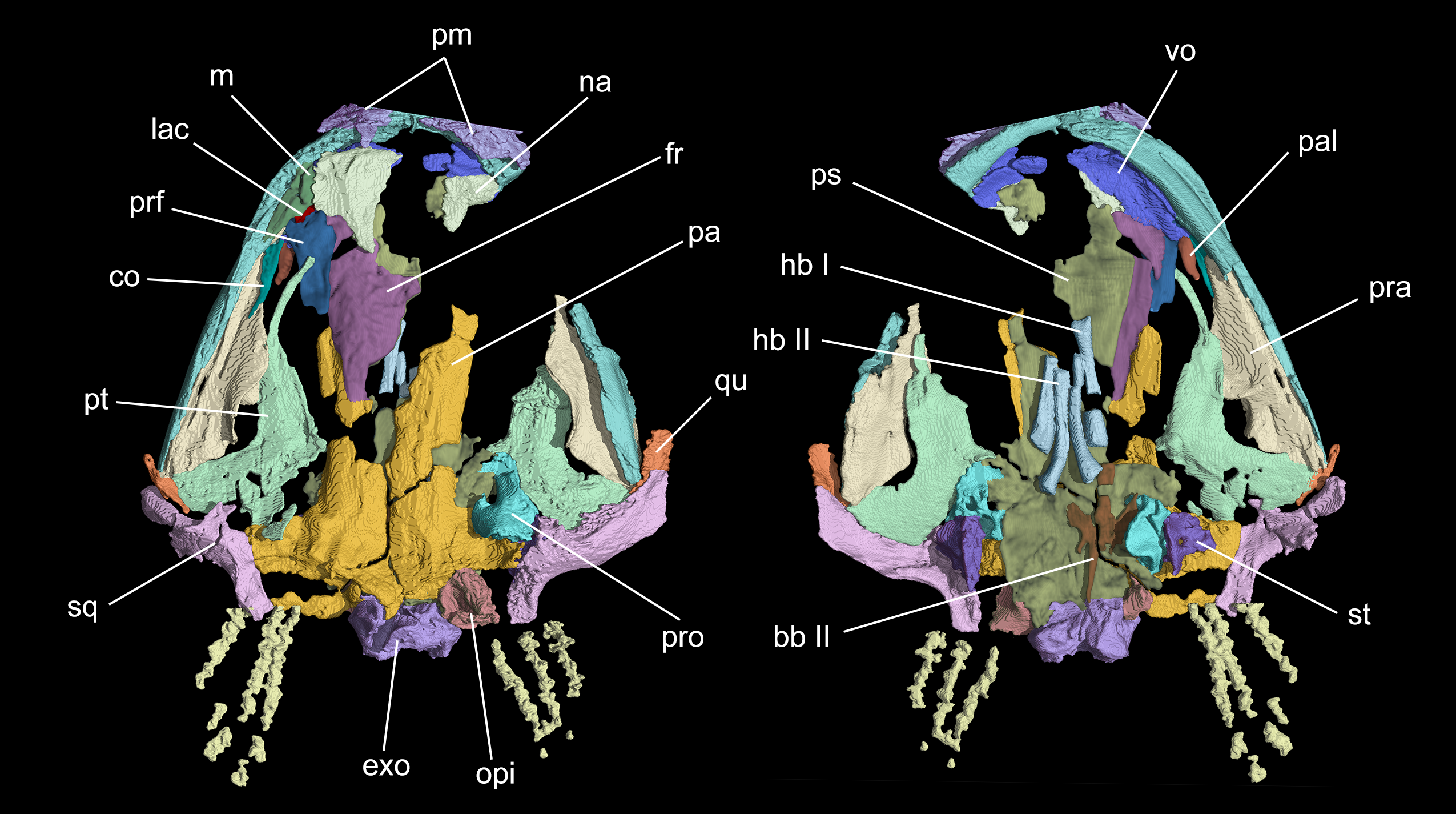

Supplement: S1 Fig — Abbreviations used as in Meta Data section. (TIF) [file pone.0153834.s002.tif]

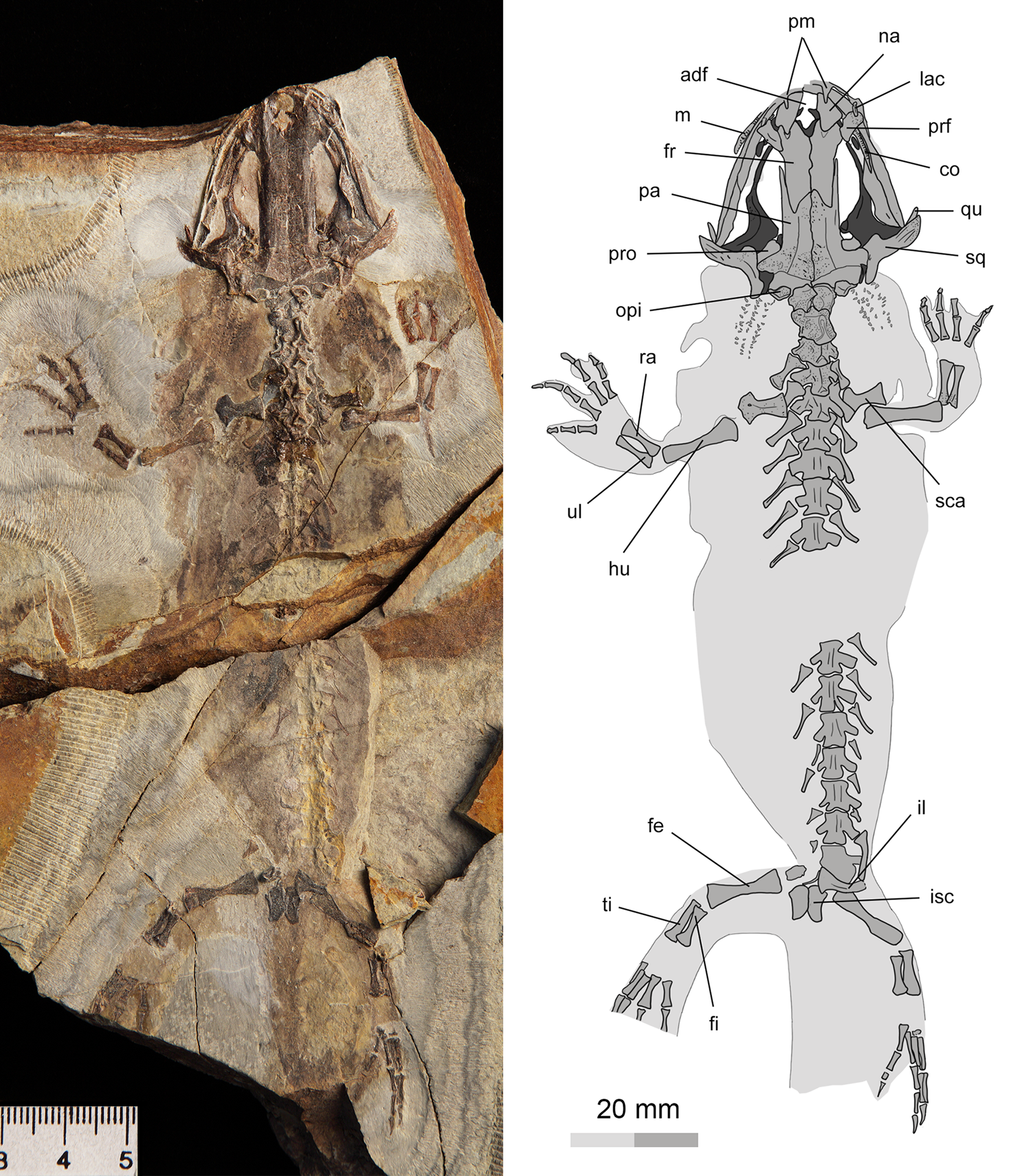

Supplement: S2 Fig — Photograph (left) and line drawing (right) of incomplete skeleton in dorsal view. Abbreviations used as in Meta Data section. (TIF) [file pone.0153834.s003.tif]

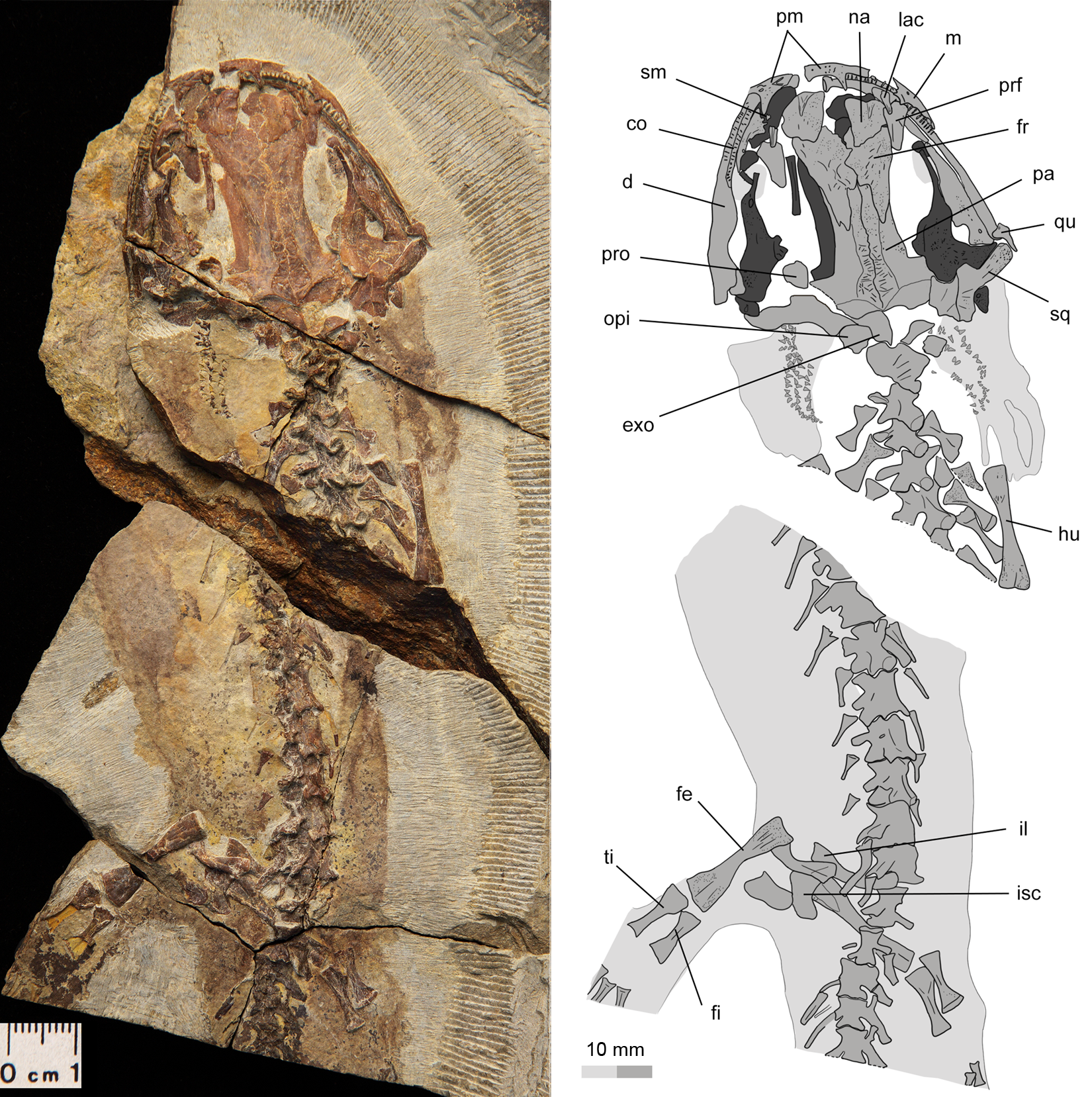

Supplement: S3 Fig — Photograph (left) and line drawing (right) of incomplete skeleton in dorsal view. Abbreviations used as in Meta Data section. (TIF) [file pone.0153834.s004.tif]

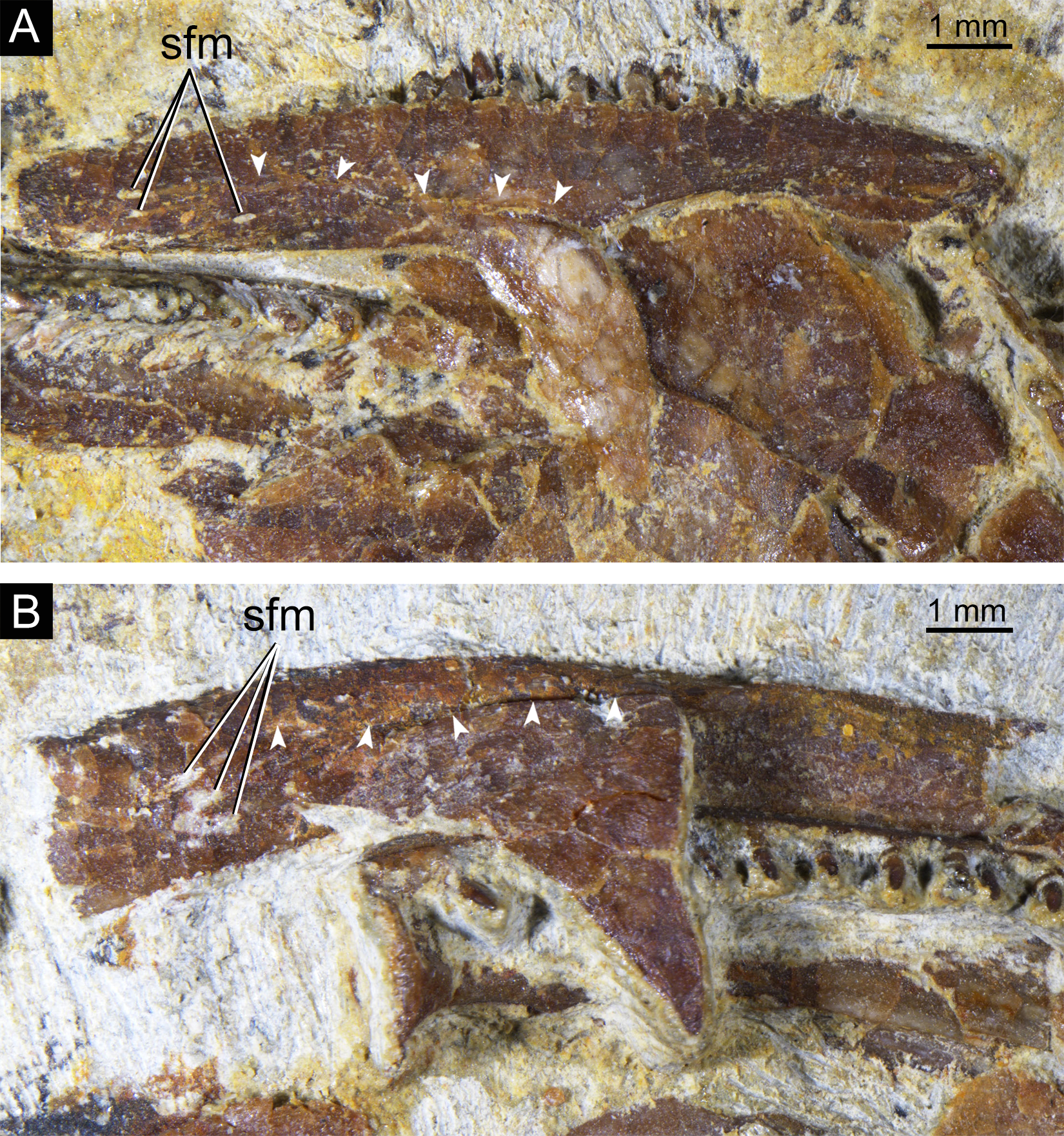

Supplement: S4 Fig — A, PKUP V0237; B, PKUP V0254. (TIF) [file pone.0153834.s005.tif]

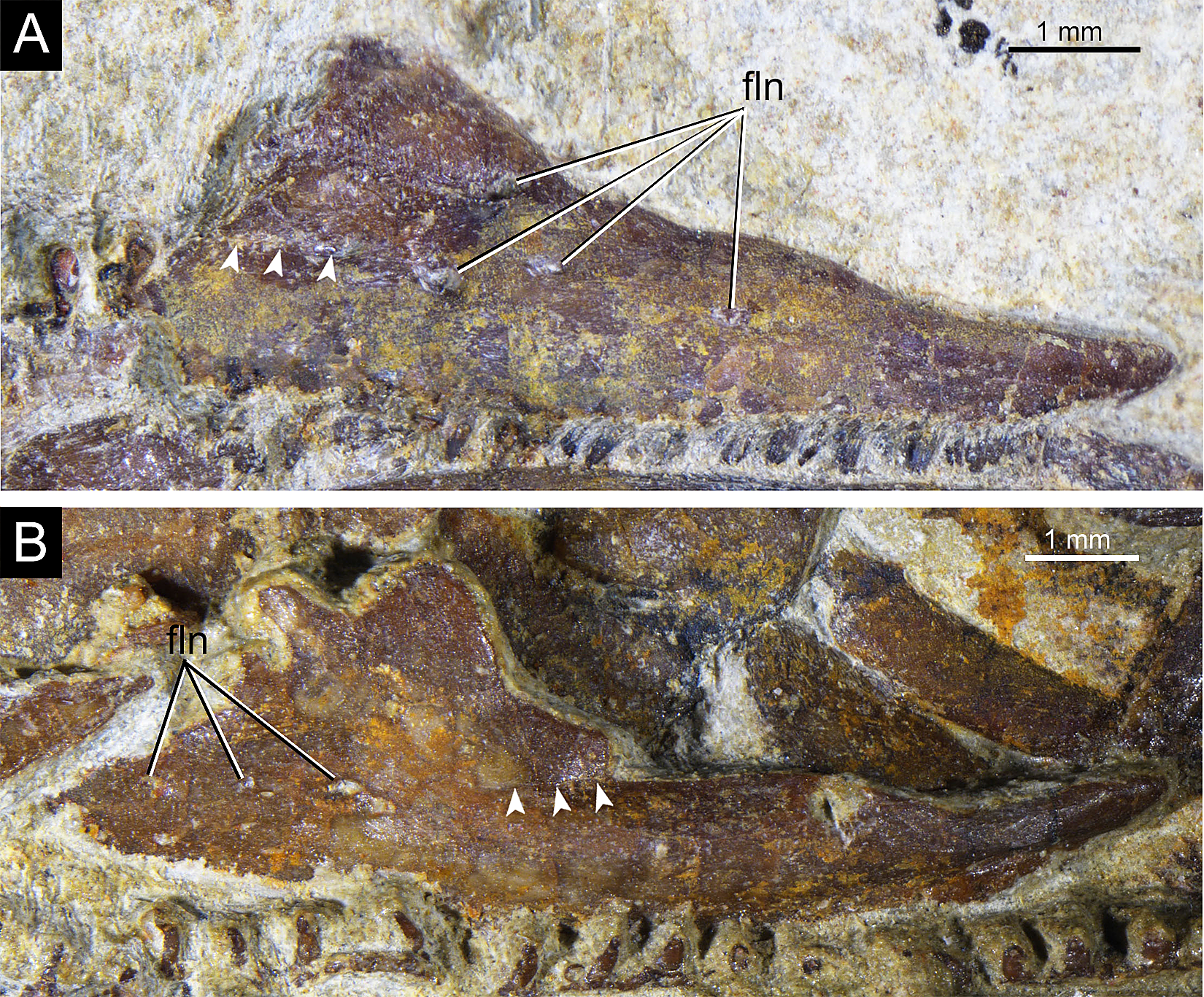

Supplement: S5 Fig — A, PKUP V0234; B, PKUP V0254. (TIF) [file pone.0153834.s006.tif]

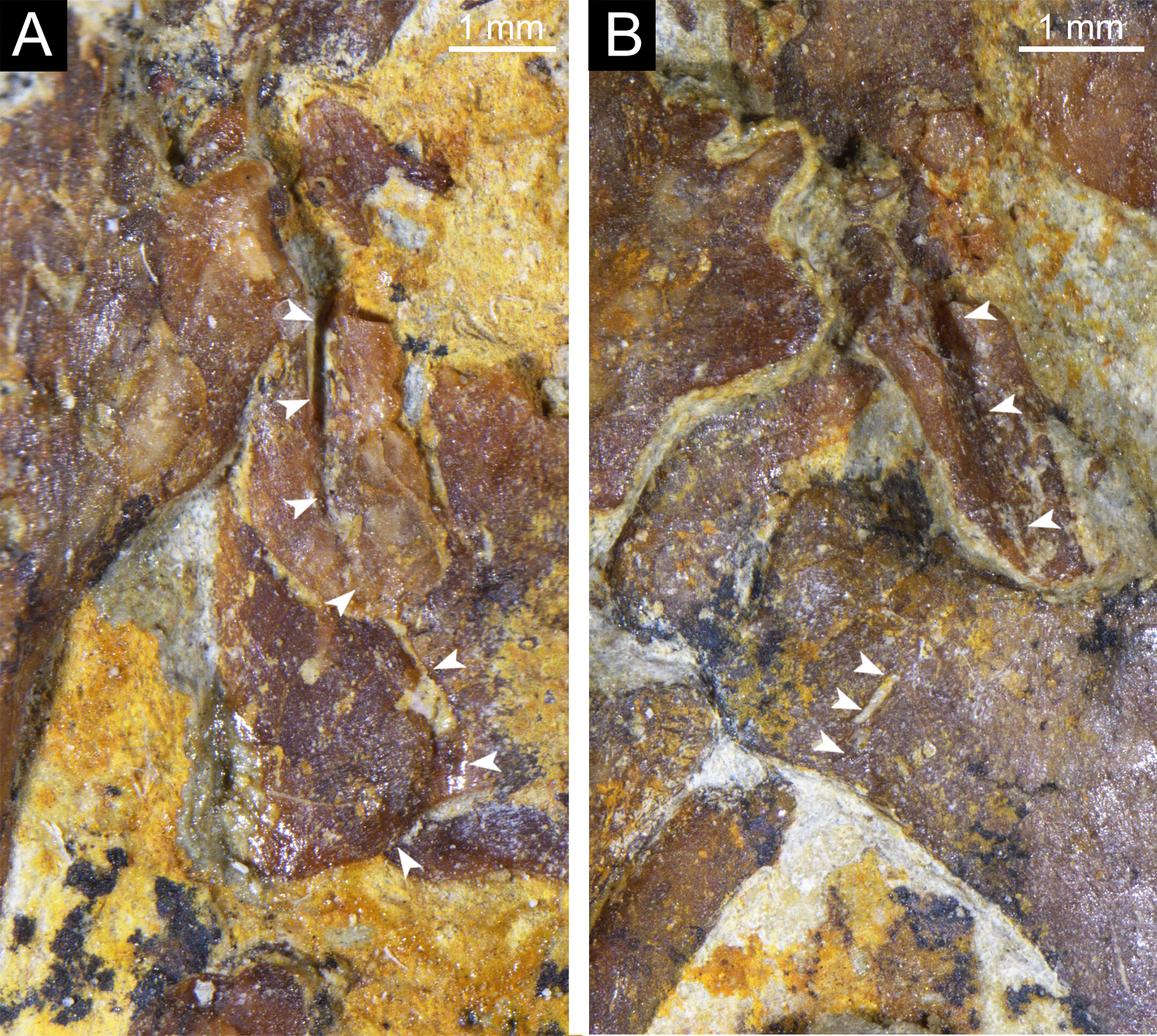

Supplement: S6 Fig — A, PKUP V0237; B, PKUP V0254. (TIF) [file pone.0153834.s007.tif]

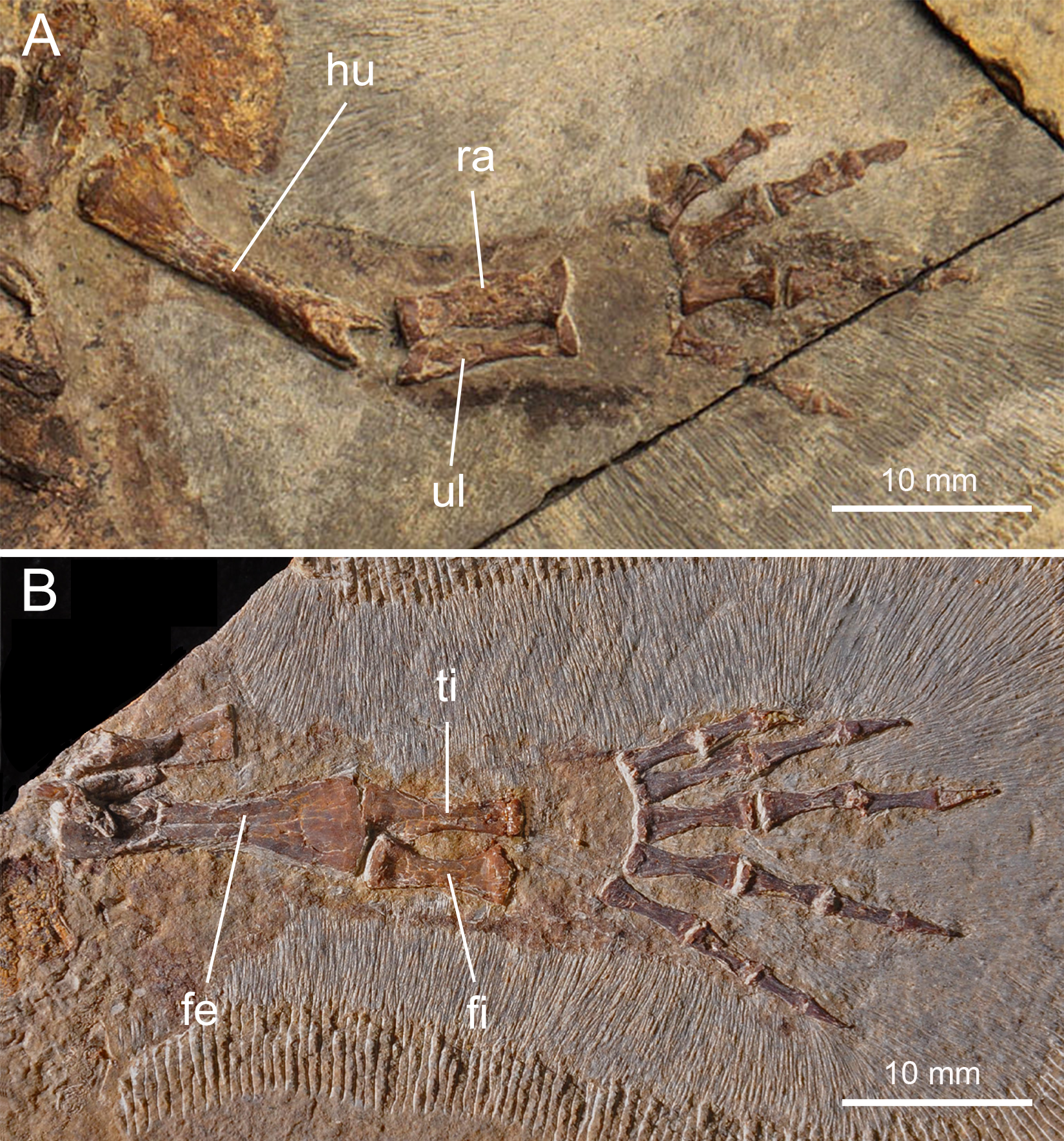

Supplement: S7 Fig — A, PKUP V0245; B, PKUP V0251. Abbreviations used as in Meta Data section. (TIF) [file pone.0153834.s008.tif]
